# *StWRKY65* stimulates thermotolerance in potato (*Solanum tuberosum* L.) through antioxidant and photosynthetic modulation

**DOI:** 10.3389/fpls.2025.1634338

**Published:** 2025-07-21

**Authors:** Xi Zhu, Xiaoqin Duan, Junfu Luo, Nengkang Guan, Haifei Zheng, Yasir Majeed, Huafen Zou, Hui Jin, Zhuo Chen, Yu Zhang

**Affiliations:** ^1^ Key Laboratory of Tropical Fruit Biology, Ministry of Agriculture and Rural Affairs/Key Laboratory of Hainan Province for Postharvest Physiology and Technology of Tropical Horticultural Products, South Subtropical Crops Research Institute, Chinese Academy of Tropical Agricultural Sciences, Zhanjiang, Guangdong, China; ^2^ National Key Laboratory for Tropical Crop Breeding, Sanya Research Institute, Chinese Academy of Tropical Agricultural Sciences, Sanya, China; ^3^ State Key Laboratory of Aridland Crop Science, Gansu Agricultural University, Lanzhou, China; ^4^ College of Agronomy, Gansu Agricultural University, Lanzhou, China; ^5^ College of Tropical Crops, Yunnan Agricultural University, Pu’er, China

**Keywords:** StWRKY65, *Solanum tuberosum*, heat stress, antioxidant enzymes, photosynthesis

## Abstract

Heat stress severely impacts the growth and development of potato plants. However, the molecular mechanisms underlying thermotolerance, particularly the role of WRKY transcription factors (TFs), remain poorly understood. Here, we identified *StWRKY65* as a heat-responsive gene in potato, demonstrating significant transcriptional upregulation under 30°C and 35°C heat stress conditions. Phylogenetic analysis classified StWRKY65 into the WRKY Group II family, revealing high evolutionary conservation with its tomato ortholog SlWRKY65. Subcellular localization confirmed its nuclear targeting. Additionally, we generated transgenic potato lines with overexpression (OE) or RNA interference (RNAi)-mediated suppression of the target gene. Under heat stress, OE lines exhibited enhanced thermotolerance, manifested through improvements in plant height, fresh/dry weights of plants and roots, tuber yield, photosynthetic efficiency, transpiration rate, and stomatal conductance. Concurrently, compared to non-transgenic (NT) controls, *StWRKY65* overexpression significantly elevated the activities of antioxidant enzymes (ascorbate peroxidase [APX], catalase [CAT], peroxidase [POD], superoxide dismutase [SOD]), proline accumulation, and total chlorophyll content, while reducing malondialdehyde (MDA) and hydrogen peroxide (H_2_O_2_) levels. Conversely, RNAi lines displayed heightened heat sensitivity, impaired growth parameters, diminished antioxidant capacity, and elevated oxidative stress markers. Furthermore, *StWRKY65* overexpression transcriptionally activated key antioxidant enzyme-related genes (*StAPX*, *StCAT1/2*, *StPOD12/47*, *StFeSOD2/3*, *StMnSOD*, *StCuZnSOD1/2*), whereas its knockdown produced opposing effects. This study demonstrates the pivotal role of *StWRKY65* in enhancing potato thermotolerance by simultaneously boosting antioxidant defenses and maintaining photosynthetic efficiency under heat stress. As climate change intensifies thermal extremes, these findings position *StWRKY65* as a valuable genetic target for developing heat-resistant potato varieties, offering a timely strategy to protect this vital crop and global food security.

## 1 Introduction

Potatoes are a vital food crop due to their nutritional value and adaptability. They provide plant-based protein and can serve as a replacement for animal-based protein, making them a key staple food in climate change and population growth (Devaux et al., 2020). Potatoes are the world’s fourth-largest food crop after rice, wheat, and maize, valued for their high yield and nutritional benefits (Kumar et al., 2021). Global potato production has increased by about 20% since 1990, but it remains below wheat, maize, and rice. Its high yield per unit area has boosted its global popularity (FAOSTAT, 2023; Jennings et al., 2020). The impacts of climate change on potato production are complex, with studies yielding varied results. Elevated temperatures may reduce yields by accelerating development rates and increasing respiration (Haverkort and Verhagen, 2008). High temperatures (>30°C) severely impair potato growth and yield, causing physical tuber damage, reduced starch content, and premature sprouting (Timlin et al., 2006). As temperate crops, potatoes thrive at 16–19°C but tolerate 25–30°C (Aien et al., 2017). Temperatures exceeding 30°C halt developmental processes by inducing stomatal closure. Multiple studies confirm that heat stress disproportionately affects potato productivity compared to other climatic factors (Adavi et al., 2018; Jennings et al., 2020; Zhang et al., 2021; Chaudhary et al., 2023; Wang et al., 2024; Bashir et al., 2025). Plants employ adaptive mechanisms, including stress-responsive TFs like MYB, AP2/ERFBP, NAC, and WRKY, to mitigate high-temperature damage. Among these, WRKY TFs play a pivotal role in regulating pathways that combat diverse abiotic stresses, such as heat, drought, and salinity (Liu et al., 2021a).

TFs are key regulators in plant signaling, controlling responses to biotic/abiotic stresses and internal developmental signals. WRKY TFs, named after their DNA-binding motif, are one of the largest plant-specific TF families. They modulate signal transduction pathways, influencing nutrient deficiency, embryogenesis, seed development, senescence, hormone-regulated processes, and responses to abiotic stressors (Wani et al., 2021). The WRKY protein structure consists of an N-terminal DNA-binding domain and a C-terminal zinc-finger motif (Xu et al., 2020). The DNA-binding domain typically contains the conserved heptapeptide WRKYGQK, though variants (e.g., WRKYGKK, WSKYGQK) exist (Meher et al., 2023). The zinc-finger motif is primarily C_2_H_2_ or C_2_HC, with atypical forms (e.g., CX_29_HXH, CX7CX_24_HXC) also reported (Zhang et al., 2024a). Based on domain number and zinc-finger type, WRKY TFs are classified into three groups: Group I: Two WRKY domains + C_2_H_2_ zinc-finger, Group II: Single WRKY domain + C_2_H_2_ zinc-finger, further subdivided into IIa–IIe phylogenetically, and Group III: Single WRKY domain + C_2_HC zinc-finger (Mahiwal et al., 2024). Evolutionary analyses suggest Group II is polyphyletic, with distinct clades (I, IIa+IIb, IIc, IId+IIe, III) (Rinerson et al., 2015). Some WRKY proteins also feature additional domains, such as glutamate/proline-rich regions and leucine zippers, which may influence function (Chen et al., 2012). Previous studies demonstrated that the WRKY TFs play key roles in plant abiotic stress responses, such as in *Arabidopsis thaliana*, high temperature suppresses *AtWRKY33* but induces *AtWRKY25*/*26*, and their overexpression enhances heat resistance (Li et al., 2011). Similarly, *AtWRKY39* (Group II) is activated by heat and regulated by SA/JA signaling pathways, with its overexpression improving thermotolerance (Li et al., 2010). In tobacco (*Nicotiana tabacum*), *NtWRKY65* regulates the expression of genes related to salt stress (Zhang et al., 2024b). In wheat (*Triticum aestivum*), *TaWRKY33* overexpression improves heat tolerance, demonstrating WRKY TFs’ diverse roles in stress responses (He et al., 2016). In maize (*Zea mays*), the overexpression of *ZmWRKY40* regulates drought tolerance in transgenic *Arabidopsis* (Wang et al., 2018a), and *ZmWRKY106* confers drought and heat tolerance in transgenic maize (Wang et al., 2018b). In rice (*Oryza sativa*), *OsWRKY11* enhanced drought tolerance (Lee et al., 2018). In tomato (*Solanum lycopersicum*), *SlWRKY80* plays a crucial role in enabling transgenic tomato plants to tolerate saline-alkali stress more effectively than wild-type plants (Shang et al., 2024). In pepper (*Capsicum annuum*), *CaWRKY40* enhances thermotolerance, with overexpression in tobacco improving heat resistance while its loss reduces tolerance (Liu et al., 2021b). However, the specific functions and regulatory mechanisms of potato WRKY family members (especially *StWRKY65*) in heat stress response have not yet been elucidated.

Despite the identification of 79 WRKY genes in potato (Zhang et al., 2017) and the characterization of several members (e.g., *StWRKY19/25/29*) in abiotic stress responses (Jiang et al., 2025), the specific role of *StWRKY65* in heat stress tolerance remains completely unknown. This represents a critical knowledge gap because WRKY TFs are master regulators of plant stress responses, yet potato WRKY networks are poorly understood. Heat stress is becoming increasingly detrimental to potato yields under climate change, and preliminary evidence suggests *StWRKY65* may have unique functional properties distinct from other stress-responsive WRKYs. Our study is the first to systematically investigate *StWRKY65’*s involvement in heat stress adaptation, which could reveal novel mechanisms of thermotolerance in this globally important crop. Understanding *StWRKY65*’s function may provide crucial molecular targets for developing heat-resistant potato varieties through either conventional breeding or biotechnological approaches. Focusing on the *StWRKY65* gene, this study employs a multi-faceted approach, integrating sequence alignment and phylogenetic analysis, expression dynamics, subcellular localization, and transgenic assays to unravel its contribution to thermotolerance. Our results reveal that *StWRKY65* coordinates heat adaptation by regulating growth traits (plant height, tuber yield, fresh and dry weights of plants and their roots), antioxidant defenses systems (APX, SOD, POD, CAT), photosynthetic performance (Net photosynthetic rate, transpiration rate, and stomatal conductance), and the induction of antioxidant enzymes related genes (*StAPX, StCAT1, StCAT2, StPOD12, StPOD47, StFeSOD2, StFeSOD3, StMnSOD, StCuZnSOD1*, and *StCuZnSOD2*). These findings uncover a pivotal mechanism underlying *StWRKY65*-mediated stress resilience, offering a genetic toolkit for climate-smart potato breeding.

## 2 Materials and methods

### 2.1 Plant material and experimental setup

The heat-sensitive potato cultivar ‘Atlantic’ (Ahn et al., 2004; Gautam et al., 2024) and the heat-tolerant cultivar ‘Désirée’ (Basu and Minhas, 1991; Ahn et al., 2004) were selected as model genotypes for studying heat stress responses. Apical buds from tissue-cultured seedlings of two potato cultivars, ‘*Atlantic*’ and ‘*Desiree*’, were initially grown on MS medium for 28 days under controlled conditions (16-hour light/8-hour dark cycle, 2800 lux light intensity, 20°C). Afterward, the seedlings were transferred to autoclaved substrate soil and grown for an additional 14 days in a growth chamber under the same photoperiod and temperature but with 75% relative humidity. Healthy, uniform plants were then potted in a soil-vermiculite mix (1:1 v/v) and maintained at 70–75% soil moisture for five weeks. To study the role of the *StWRKY65* gene under abiotic stress, plants were subjected to drought stress (10% and 20% PEG6000), heat stress (30°C and 35°C), and salt stress (75 mM and 150 mM NaCl). Samples were collected at 0, 1, 3, 6, 12, 24, and 48 h post-treatment for gene expression analysis. The experiment involved 432 plants, accounting for two cultivars (‘*Atlantic*’ and ‘*Desiree*’), three replicates per treatment, and multiple time points. For physiological and photosynthetic traits assessments under heat stress, transgenic and NT lines of both cultivars were grown similarly for five weeks before exposure to 30°C and 35°C for 48 hours. A total of 378 plants were analyzed, including NT controls, *StWRKY65* overexpression lines, and RNAi-silenced lines, each with three biological and technical replicates.

### 2.2 Multiple-sequence alignment and phylogenetic analysis

The amino acid sequence of potato *StWRKY65* was aligned with its orthologs from 8 other plant species using CLUSTALW (v1.83) (Thompson et al., 1994) to identify conserved motifs. A phylogenetic tree was then constructed using MEGA X (v4.1) (Dereeper et al., 2010) with the Neighbor-Joining (NJ) method. Branch reliability was evaluated through 1000 bootstrap replicates to ensure robust nodal support.

### 2.3 RNA extraction and qPCR analysis

According to the manufacturer’s instructions, total RNA was isolated from plant samples using TRIzol reagent (Invitrogen, USA). Subsequently, 1 µg of total RNA was reverse-transcribed into cDNA using the TransScript First-Strand cDNA Synthesis Kit (TransGen Biotech, China). Quantitative real-time PCR (qRT-PCR) was performed on a LightCycler 480 II system (Roche, Switzerland) in a 20 µL reaction volume containing 100 ng cDNA, 0.8 µL of gene-specific primers (**Supplementary Table S1**), and 10 µL of SYBR Premix Ex Taq (2×) (Takara, Japan). The thermal cycling conditions included initial denaturation at 94°C for 2 minutes, 34 cycles of denaturation at 94°C for 30 seconds, annealing at 60°C for 34 seconds, and extension at 72°C for 30 seconds. The *Stef1α* gene served as an internal reference for normalization, and relative gene expression was calculated using the 2^−ΔΔCt^ method (Livak and Schmittgen, 2001). Three biological replicates, each with three technical replicates, were analyzed to ensure data reliability. Primer sequences used during this study are listed in **Supplementary Table S1**.

### 2.4 Plasmid construction and genetic transformation

The coding sequence of *StWRKY65* (GenBank ID: XM_006348830.2) was amplified via PCR using gene-specific primers (forward: 5’-CTCGACATGGAAGATAGTCTATACAA-3’; reverse: 5’-GTCGACTCCTGTACCGCCGCAGCAGG-3’) and subsequently inserted into the pBI121-EGFP vector using standard cloning techniques (Li et al., 2020). For RNA interference studies following the previous method (Lu et al., 2019), the sense strand cDNA fragment was amplified with *Eco*R I/*Kpn* I restriction sites primers and ligated into the pHANNIBAL vector (pHAN-StWRKY65-R). The antisense fragment was similarly amplified using *Hind* III/*Xba* I primers and cloned to generate pHAN-StWRKY65-RF. This construct was then transferred into the pART vector at *Xho* I and *Xba* I sites, yielding the final pART-StWRKY65-RNAi vector. The recombinant plasmid was introduced into *Agrobacterium tumefaciens* strain LBA4404 and cultured at 28°C for 48 hours. Transformed colonies were verified through PCR using RNAi-specific primers (forward: 5’-CGATGCAGTAGCTCCAAAGGCT-3’; reverse: 5’-TGAAAATC CATTATCGTGTTGAT-3’) and *NPT II* selection marker primers (forward: 5’-ATGACTGGGC ACAACAGACAATCG-3’; reverse: 5’-TCAGAAGAACTCGTCAAGAAGGCG-3’). Bacterial cultures were maintained in an LB medium containing 50 mg/L each of spectinomycin and gentamicin.

For plant transformation, *Agrobacterium* cells were pelleted by centrifugation at 5,000 rpm for 10 minutes and resuspended in MS liquid medium to an optical density of OD_600_ = 0.3. Sterilized potato stem segments (2 cm) were pre-cultured on MS medium supplemented with 7.4 g/L agar, 30 g/L sucrose, and plant growth regulators (0.5 mg/L 6-BA, 2.0 mg/L ZT, 0.2 mg/L GA3, and 1.0 mg/L IAA, pH 5.8). Following a 10-minute inoculation with bacterial suspension, explants were co-cultivated in darkness at 25°C for 72 hours. Transformed tissues were then transferred to selection medium containing 300 mg/L timentin and 100 mg/L kanamycin, with sub-culturing every two weeks. Antibiotic-resistant shoots were excised and transferred to rooting medium (MS basal salts, 7.4 g/L agar, 30 g/L sucrose, 300 mg/L timentin, and 100 mg/L kanamycin) to induce root formation.

### 2.5 Subcellular localization analysis of StWRKY65 in tobacco epidermal cells

To determine the subcellular localization of StWRKY65, its coding sequence was PCR-amplified using specific primers (forward: 5′-ATGGAAGATAGTCTATACAA-3′; reverse: 5′-TCCTGTACCGCCGCAGCAGG-3′) and inserted into the pCAM35s-GFP expression vector. The resulting recombinant plasmid was transformed into *Agrobacterium tumefaciens* GV3101. For transient expression analysis, *Nicotiana benthamiana* leaves were infiltrated with the *Agrobacterium* suspension following established methods (Sparkes et al., 2006). After 48 hours of incubation, GFP fluorescence was examined using a Leica TCS SP8 confocal microscope (Leica Microsystems, Germany) to assess the subcellular distribution of the StWRKY65-GFP fusion protein.

### 2.6 Evaluation of growth parameters

To analyze growth performance under heat stress, apical meristems from both transgenic and NT potato cultivars (‘*Atlantic*’ and ‘*Desiree*’) were initially cultured on MS medium for 28 days under controlled environmental conditions (16/8-hour light/dark cycle, 2800 lux illumination, 20°C). Following this establishment phase, plantlets were transferred to sterilized growth substrate and maintained in a climate-controlled chamber for two weeks with identical light conditions at 20°C and 70% relative humidity. After selecting plants with uniform growth characteristics, specimens were potted (26 × 27 × 18 cm containers) in a 1:1 soil-vermiculite mixture and cultivated for 35 days with soil moisture regulated to 70-75% field capacity. Heat stress treatments were then implemented by exposing plants to elevated temperatures (30°C and 35°C) for six weeks. Multiple growth parameters were quantified, including plant height, tuber yield per plant, plant fresh and dry weights, and root fresh and dry weight. Fresh weight measurements were conducted immediately post-treatment, while dry weight determinations involved desiccation at 70°C until constant mass was achieved. The experimental design incorporated 378 total plants, comprising two distinct potato varieties, seven genetic lines per cultivar (NT, three *StWRKY65-*overexpression lines, and three *StWRKY65-*knockdown lines), and three temperature regimes. Three biological and three technical replications were employed throughout the experiments.

### 2.7 Evaluation of physiological parameters and chlorophyll content

The study analyzed key physiological parameters in 378 seedlings from two cultivars, each with seven genotypes: a NT control, three *StWRKY65*-overexpression lines, and three *StWRKY65*-RNAi lines. In a completely randomized design, the seedlings were subjected to two heat stress conditions (30°C and 35°C). Enzyme activities, APX POD, SOD, and CAT, were measured using standard methods (Nakano and Asada, 1981; Maehly and Chance, 1954; Giannopolitis and Ries, 1977; Aebi, 1984). The contents of proline, MDA, and H_2_O_2_ were also assessed (Bates et al., 1973; Heath and Packer, 1968; Bouaziz et al., 2015), and the chlorophyll content was measured by following a previously described protocol (Zhu et al., 2023). Detailed protocols are provided in **Supplementary Data 1**.

### 2.8 Evaluation of photosynthetic parameters

Photosynthetic parameters were measured on the third fully expanded leaf (counting from the shoot apex) between 9:30-11:30 AM to ensure consistent light exposure. Using a Li-COR 6400XT portable photosynthesis system, we quantified three key gas exchange parameters, comprising net photosynthetic rate, transpiration rate, and stomatal conductance. All measurements were performed under standardized conditions, including light intensity: 1500 μmol photons m^-^² s^-^¹, ambient CO_2_: 400 μmol mol^-^¹, and relative humidity: 60-70% in the leaf chamber. The study employed a completely randomized design with three biological replicates for each treatment combination.

### 2.9 Statistical analysis

Statistical analyses were conducted with GraphPad Prism (GraphPad Software, San Diego, CA, USA) and IBM SPSS Statistics 19.0 (IBM Corporation, Chicago, IL, USA). Quantitative data were expressed as means ± standard deviation (SD). Graphical representations of results, including histograms and line graphs, were generated through GraphPad Prism. Appropriate statistical tests were selected based on experimental design: one-way ANOVA followed by Tukey’s *post hoc* test or Dunnett’s T3 procedure for group comparisons, and two-way ANOVA with Sidak’s correction for multiple comparisons.

## 3 Results

### 3.1 Multiple-sequence alignments and phylogenetic tree construction

**Figure 1:**
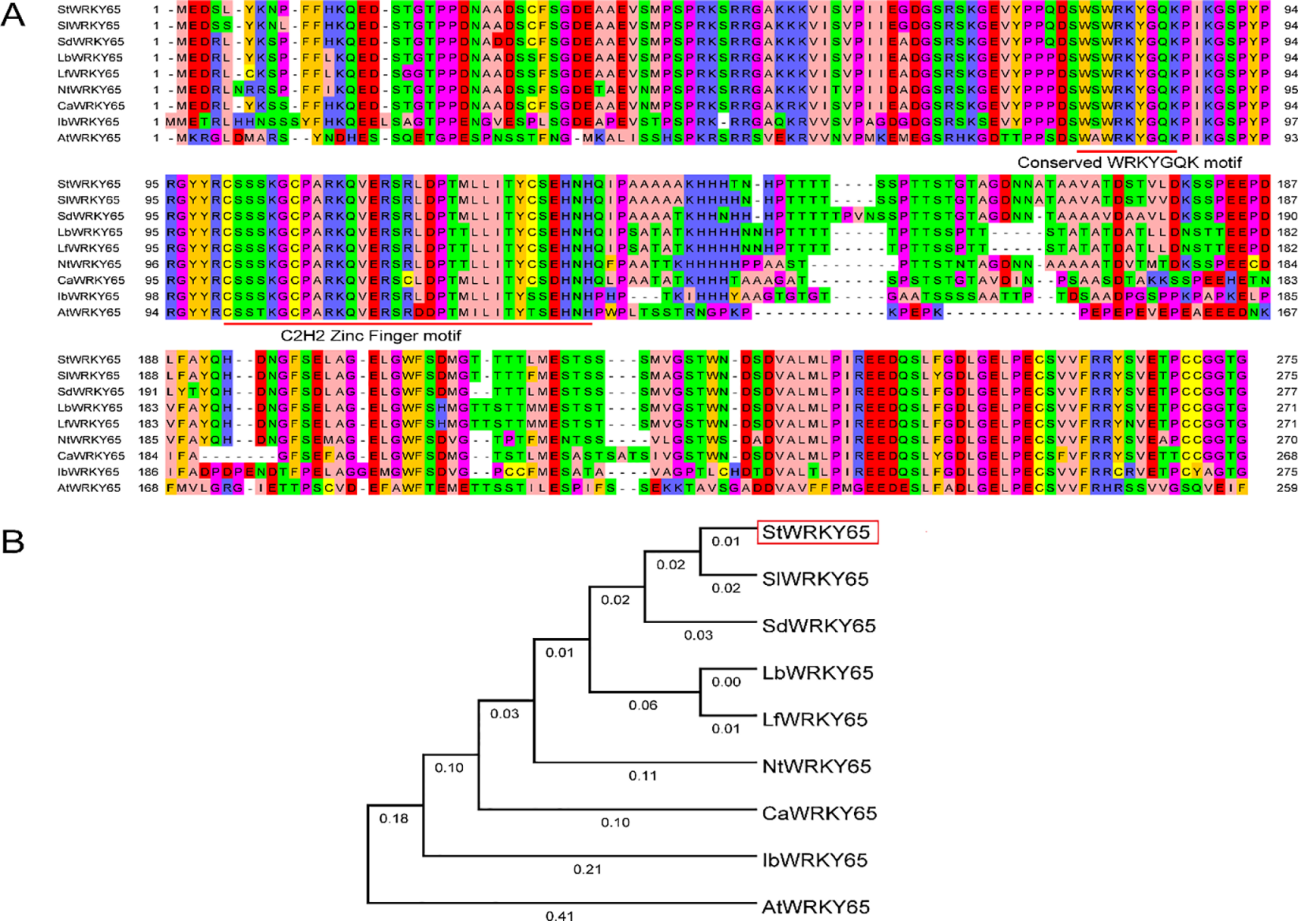
Multiple sequence alignment and phylogenetic analysis of *StWRKY65* across plant species. **(A)** Multiple sequence alignment of *WRKY65* homologs from potato and various other plant species. The background colors indicate the degree of similarity among the amino acid sequences. The first red line highlights the WRKYGQK motif, while the second marks the conserved C_2_H_2_ zinc-finger motif. **(B)** Neighbor-joining phylogenetic tree illustrating the relationships of *WRKY65* among potato and other plant species. The unrooted tree was constructed using the neighbor-joining method in MEGA 4.1. Bootstrap values exceeding 50% from 1000 replicates are shown at each branch.

The multiple-sequence alignment and subsequent phylogenetic analysis reveal the evolutionary conservation of StWRKY65 across nine divergent plant species. Potato (*Solanum tuberosum*) StWRKY65, tomato (*Solanum lycopersicum*) SlWRKY65, bittersweet nightshade *Solanum dulcamara* SdWRKY65, wolfberry (*Lycium barbarum*) LbWRKY65, African boxthorn (*Lycium ferocissimum*) LfWRKY65, tobacco (*Nicotiana tobacum*) NtWRKY65, pepper (*Capsicum annuum*) CaWRKY65, sweet potato (*Ipomoea batatas*) IbWRKY65, and *Arabidopsis* (*Arabidopsis thaliana*) AtWRKY65; their predicted amino acid residue alignment is shown in **Figure 1A**. Orthologs of WRKY65 were identified using the NCBI Protein BLAST tool (https://blast.ncbi.nlm.nih.gov/Blast.cgi?PAGE=Proteins), and their corresponding protein accession numbers are provided in **Supplementary Table S1**. Comparative sequence analysis demonstrated that StWRKY65 exhibited high evolutionary conservation with its orthologs among two species, including *Solanum lycopersicum* (SlWRKY65) and *Solanum dulcamara* (SdWRKY65). Remarkably, all identified proteins harbor the canonical WRKYGQK domain at the N-terminus, coupled with a C_2_H_2_ zinc-finger motif (CX_5_-C-X_23_-H-X_1_-H), confirming their classification within Group II of the WRKY family (**Figure 1A**). The invariant domain architecture highlights strong evolutionary conservatism in functional motifs. Phylogenetic analysis resolved WRKY65 orthologs into well-defined clusters (**Figure 1B**), with StWRKY65, SlWRKY65, and SdWRKY65 clustered closely, indicative of their high sequence similarity (>85% identity). Critically, each cluster maintained conserved domain structures and sequence homology, implying functional conservation among evolutionarily related orthologs.

### 3.2 Expression analysis of *StWRKY65* in potato under abiotic stress

**Figure 2:**
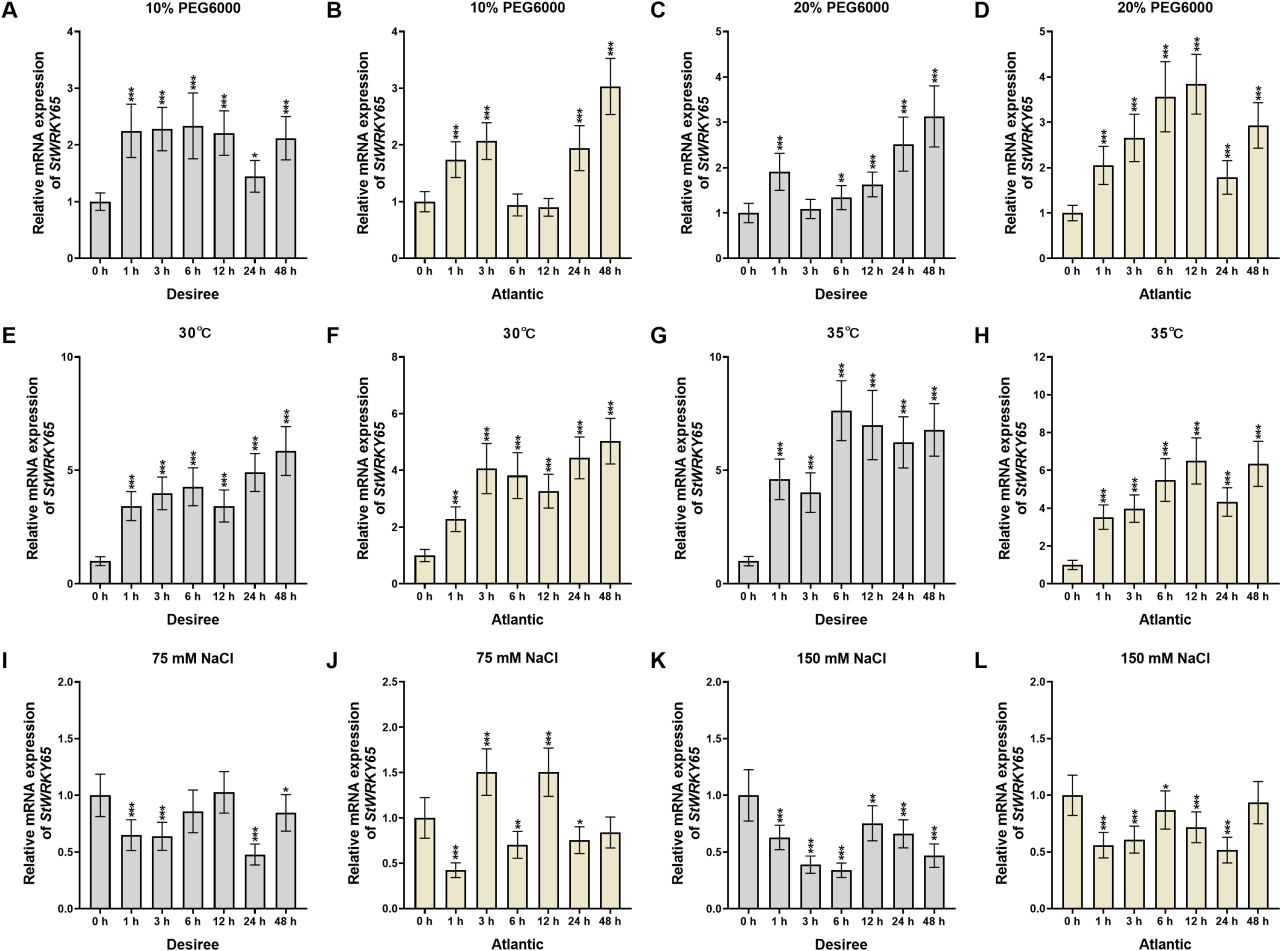
The expression patterns of *StWRKY65* in the leaves of potato cultivar ‘*Atlantic*’ and ‘*Desiree*’ in response to drought (10% and 20% PEG6000), heat (30°C and 35°C), and salinity (75 mM and 150 mM NaCl), induced stress at various time intervals (0, 1, 3, 6, 12, 24, and 48 h). The data are presented as mean ± standard deviation. P-values (*P < 0.05, **P < 0.01, ***P < 0.001) were calculated through ordinary two-way ANOVA followed by Tukey’s multiple comparisons test with a sample size of n = 9.

This study systematically analyzed the expression profiles of the *StWRKY65* gene in leaves of two potato cultivars (‘*Desiree*’ and ‘*Atlantic*’) under various abiotic stress conditions (**Figures 2A–L**). Under drought stress, *StWRKY65* exhibited expression patterns correlated with stress severity and cultivar specificity. In ‘*Desiree*’, 10% PEG6000 treatment induced a stable upregulation (with relatively steady increases at 1, 3, 6, 12, and 48 h post-treatment, peaking at 6 h), whereas 20% PEG6000 triggered fluctuating upregulation (rapid induction at 1 h, followed by a decline at 3 h, then gradual increases at 6, 12, and 24 h, reaching a peak at 24 h) (**Figures 2A–C**), suggesting its positive regulatory role under severe drought. In ‘*Atlantic*’, 10% PEG6000 elicited oscillatory expression (upregulation at 1, 3, 24, and 48 h but downregulation at 6 and 12 h), while 20% PEG6000 induced variable upregulation (peaking at 12 h) (**Figures 2B–D**), indicating its potential involvement in drought resistance.

The expression profiles of *StWRKY65* under high-temperature stress demonstrated robust and rapid induction (**Figures 2E–H**). In ‘*Desiree*’, 30°C treatment caused differential upregulation (gradual increases at 1, 3, and 6 h, a decline at 12 h, followed by rises at 24 and 48 h, peaking at 48 h), whereas 35°C elicited stronger upregulation throughout the time course (particularly from 6 to 48 h), with a rapid response peaking at 6 h (**Figures 2E–G**). This suggests that *StWRKY65* is temperature-responsive and may play a critical role in thermotolerance. In contrast, ‘*Atlantic*’ displayed distinct dynamics: at 30°C, rapid transcriptional induction occurred at 1 and 3 h, followed by declines at 6 and 12 h, then gradual increases to a peak at 48 h; under 35°C, sustained upregulation was observed at 1, 3, 6, and 12 h, a decline at 24 h, and a peak at 48 h (**Figures 2F–H**). These results indicate that *StWRKY65* is similarly induced by heat stress in ‘*Atlantic*’, likely contributing to thermal adaptation. The cultivar-specific patterns may reflect divergent evolutionary adaptations to heat stress.

Under salt stress, ‘*Desiree*’ exhibited fluctuating downregulation with 75 mM NaCl treatment (significant declines at 1 and 3 h, recovery at 6 and 12 h, a sharp drop at 24 h, and partial recovery at 48 h, reaching the lowest level at 24 h). High salinity (150 mM NaCl) caused more pronounced downregulation (continuous declines at 1, 3, and 6 h, a slight rebound at 12 h, followed by further decreases at 24 and 48 h, with the lowest level at 6 h) (**Figures 2I–K**). In ‘*Atlantic*’, 75 mM NaCl triggered a biphasic response: initial downregulation (lowest at 1 h), followed by upregulation (peak at 3 h), then declines at 6 h and rebounds at 12 h, before significant downregulation at 24 and 48 h (**Figure 2J**). Meanwhile, 150 mM NaCl predominantly induced downregulation (notably at 1, 3, 6, 12, and 24 h, with recovery at 48 h, lowest at 24 h) (**Figure 2L**), though the suppression was less severe than with 75 mM NaCl (**Figures 2I–L**). These findings highlight cultivar-specific transcriptional responses of *StWRKY65* to NaCl, suggesting its potential negative regulatory role in salt tolerance. Collectively, *StWRKY65* displayed markedly divergent expression patterns in ‘*Desiree*’ and ‘*Atlantic*’ across all stress conditions, underscoring how genetic differences between potato cultivars influence gene expression dynamics in response to environmental stresses.

### 3.3 Subcellular localization analysis of StWRKY65

**Figure 3:**
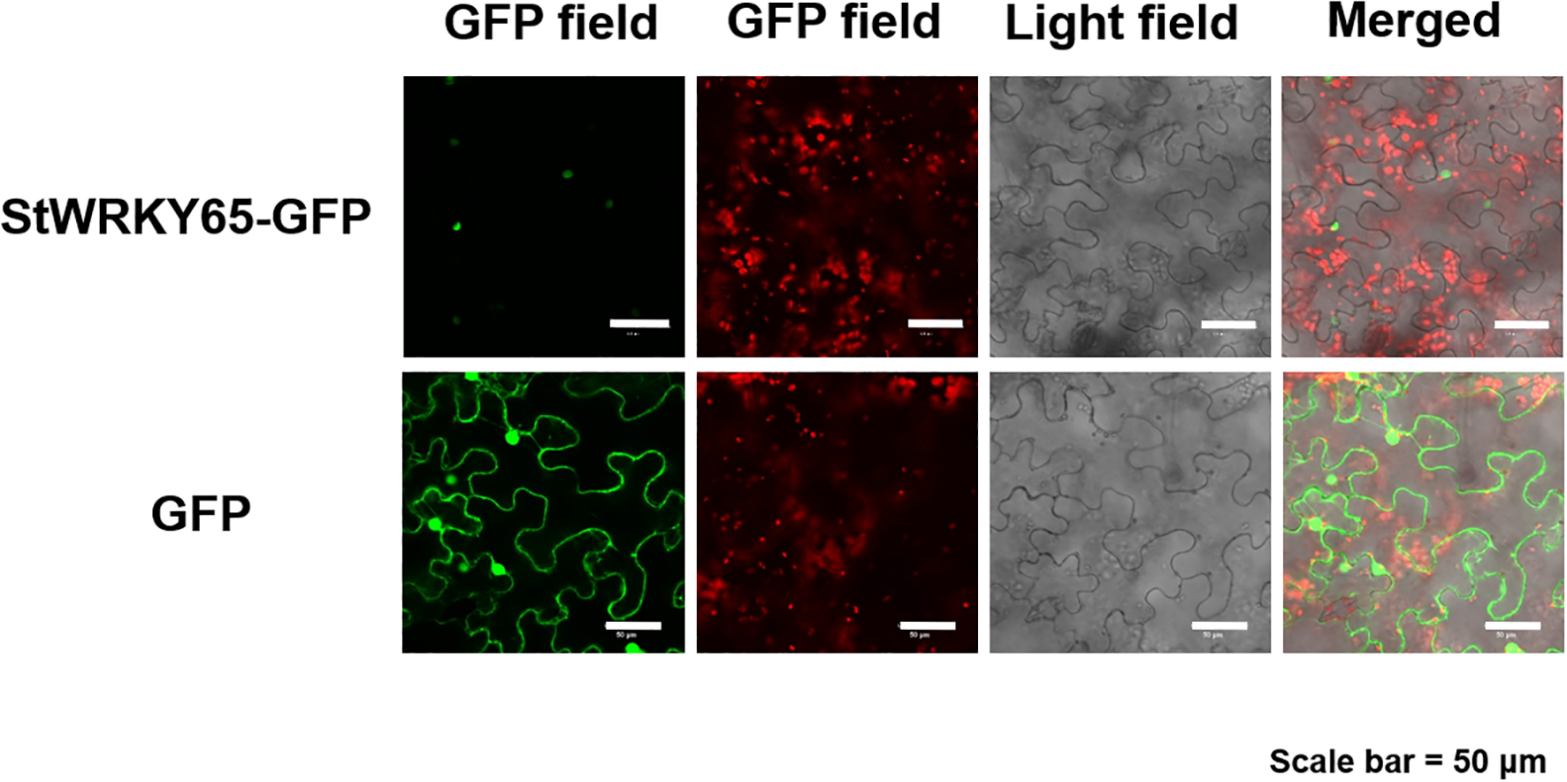
Subcellular Localization of StWRKY65-GFP Fusion Protein. Confocal laser scanning microscopy was conducted to analyze tobacco plants transformed with the pCAM35-GFP-StWRKY65 construct. The empty vector expressing only GFP was used as a control. Scale bar = 50 μm.

To elucidate the subcellular localization of StWRKY65, its coding sequence was cloned into the pCAM35S expression vector carrying the green fluorescent protein (GFP) reporter gene. The constructed StWRKY65-GFP fusion vector, along with the empty GFP vector (control), was transiently expressed in epidermal cells of *Nicotiana benthamiana* via *Agrobacterium tumefaciens* (strain GV3101)-mediated transformation. Confocal laser scanning microscopy analysis revealed distinct subcellular distribution patterns: The free GFP control exhibited fluorescence in the plasma membrane, cytoplasm, and nucleus, indicating its nonspecific cellular localization. In contrast, the StWRKY65-GFP fusion protein displayed strong green fluorescence signals exclusively in the nuclear region, with no detectable fluorescence in the cytoplasm or plasma membrane (**Figure 3**). This strict nuclear localization pattern confirms that StWRKY65 specifically resides in the nucleus, a key characteristic of functional TFs involved in transcriptional regulation. These findings provide crucial structural evidence supporting the presumed role of StWRKY65 in regulating gene expression networks within the nucleus, laying a foundation for further mechanistic studies on its biological functions.

### 3.4 Construction of *StWRKY65* transgenic potato plants

To elucidate the functional role of *StWRKY65* in heat stress responses, we generated stable transgenic potato lines with either *StWRKY65*-overexpression or RNAi-mediated knockdown in two cultivars (‘*Atlantic*’ and ‘*Desiree*’). The success of genetic transformation was confirmed by qRT-PCR analysis: OE lines exhibited 11- to 19.9-fold upregulation (***P < 0.001) of *StWRKY65* transcripts, while RNAi lines showed 54-77% suppression (***P < 0.001; **Supplementary Figure S1**). For subsequent thermotolerance assays, we strategically selected transgenic lines from each cultivar based on expression profiles: (1) three high-expression lines (‘*Atlantic*’: OE-1/2/4; ‘*Desiree*’: OE-1/3/4) (**Supplementary Figures S1A, B**) displaying maximal relative transcript accumulation of *StWRKY65*; and (2) three effective knockdown lines (‘*Atlantic*’: RNAi-1/3/4; ‘*Desiree*’: RNAi-2/3/5) (**Supplementary Figures S1C, D**) demonstrating the strongest transcriptional suppression. The rigorous selection of lines with significantly distinct genetic backgrounds provides a robust guarantee for the precise dissection of the regulatory functions of *StWRKY65* in heat stress responses.

### 3.5 *StWRKY65* modulates growth and biomass accumulation under heat stress conditions

**Figure 4:**
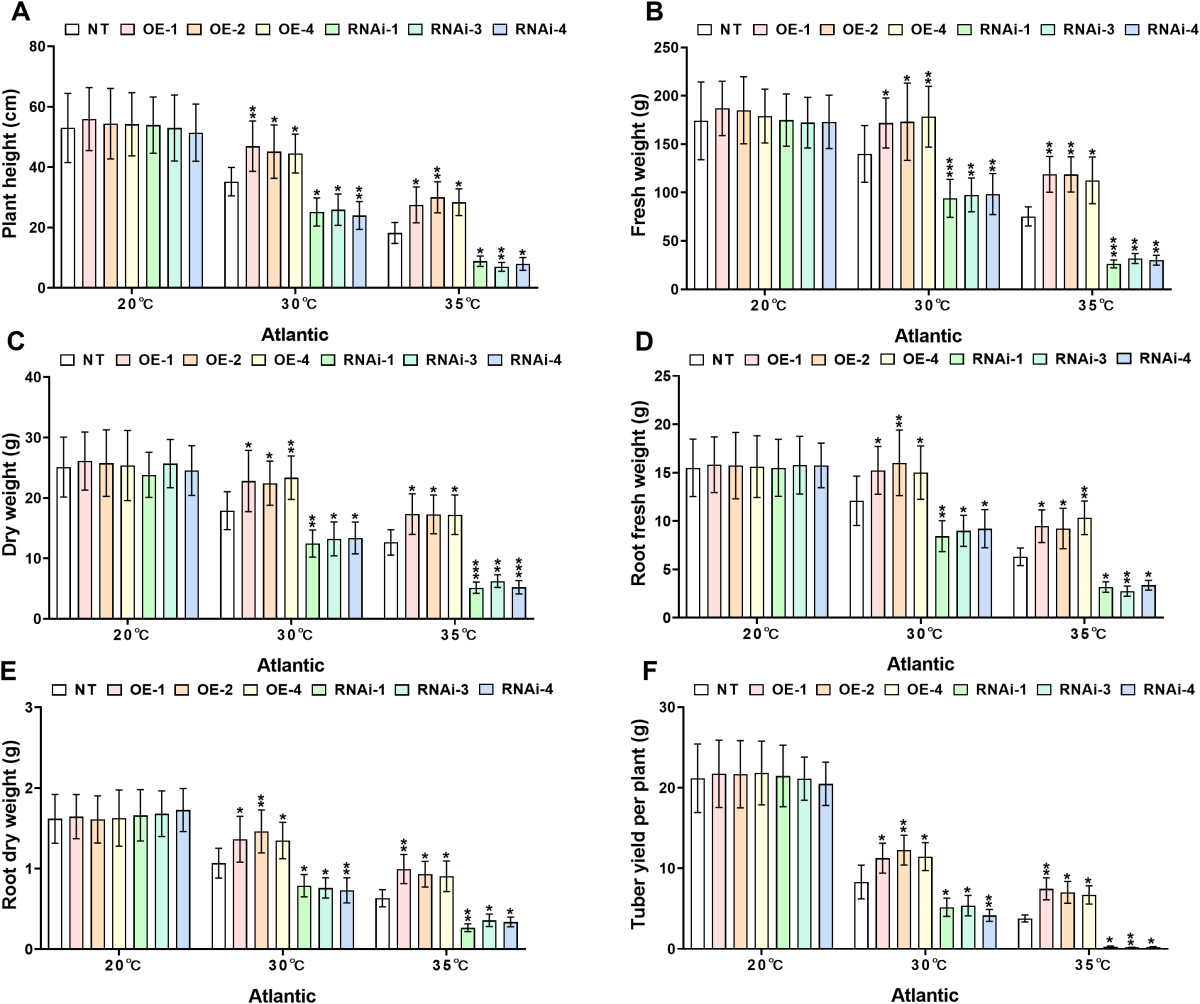
*StWRKY65* modulates potato growth parameters of cultivar ‘*Atlantic*’; **(A)** plant height, **(B)** fresh weight, **(C)** dry weight, **(D)** root fresh weight, **(E)** root dry weight, and **(F)** tuber yield per plant, after exposure to 20°C, 30°C, and 35°C of heat stress treatments. NT, non-transgenic plants; OE, pBI121-EGFP-StWRKY65-transgenic plants (OE-1, OE-2, and OE-4); RNAi, pART-StWRKY65-RNAi-transgenic plants (RNAi-1, RNAi-3 and RNAi-4). The data are presented as mean ± standard deviation. P-values (*P < 0.05, **P < 0.01) were calculated through ordinary two-way ANOVA followed by Tukey’s multiple comparisons test with a sample size of n = 9.

**Figure 5:**
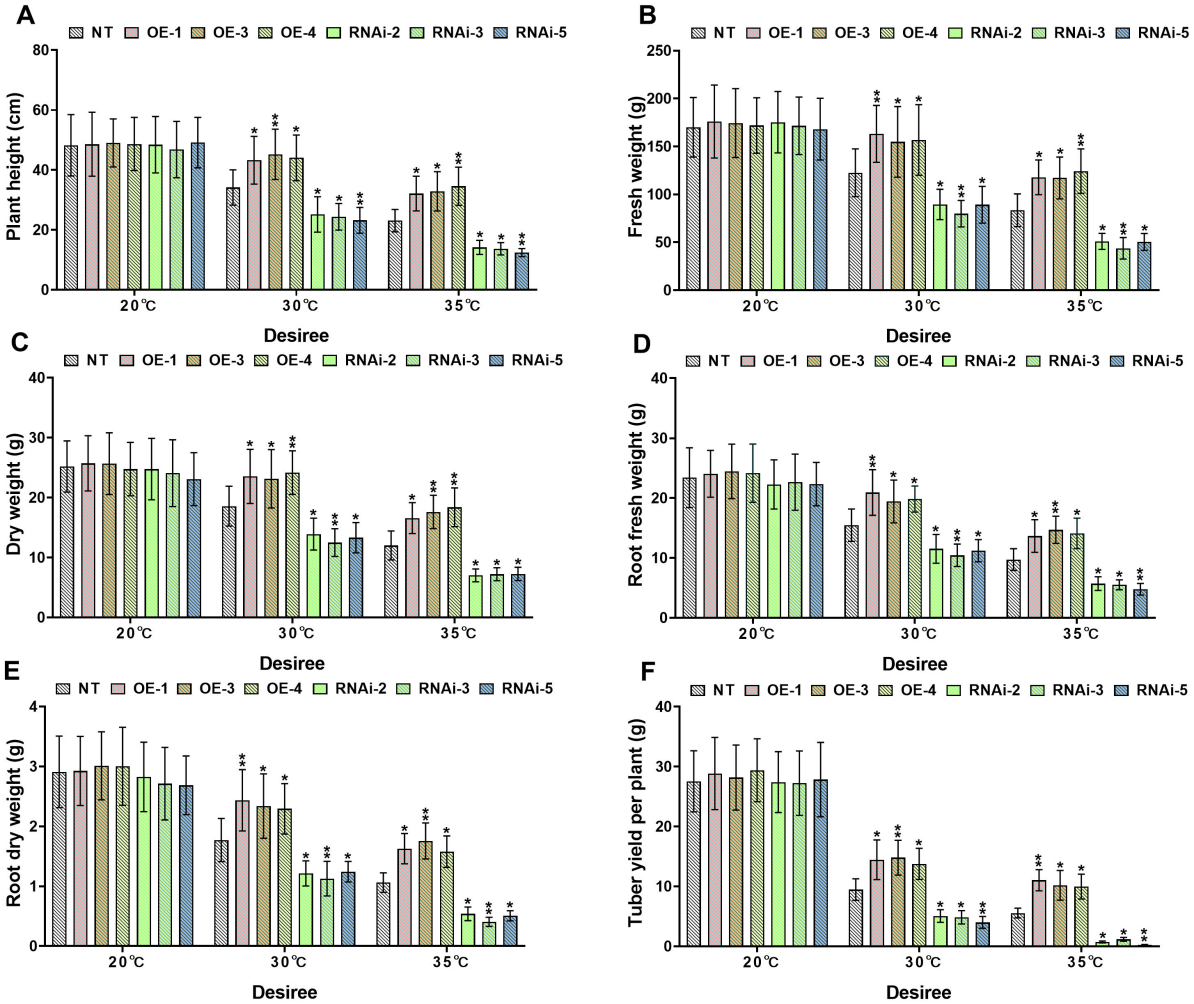
*StWRKY65* modulates potato growth parameters of cultivar ‘*Desiree*’; **(A)** plant height, **(B)** fresh weight, **(C)** dry weight, **(D)** root fresh weight, **(E)** root dry weight, and **(F)** tuber yield per plant, after exposure to 20°C, 30°C, and 35°C of heat stress treatments. NT, non-transgenic plants; OE, pBI121-EGFP-StWRKY65-transgenic plants (OE-1, OE-3, and OE-4); RNAi, pART-StWRKY65-RNAi-transgenic plants (RNAi-2, RNAi-3 and RNAi-5). The data are presented as mean ± standard deviation. P-values (*P < 0.05, **P < 0.01) were calculated through ordinary two-way ANOVA followed by Tukey’s multiple comparisons test with a sample size of n = 9.

Growth index analysis demonstrated significant differences in heat tolerance between transgenic and NT potato lines under distinct heat stress conditions (30°C and 35°C). Under control conditions (20°C), no significant differences (*P*>0.05) were detected in growth indices, including plant height, plant fresh and dry weights, root fresh and dry weights, and tuber weight, among genotypes of both the ‘*Atlantic*’ and ‘*Desiree*’ varieties, as illustrated in **Figures 4**, **5**. When subjected to 30°C heat stress, all growth indices of ‘*Atlantic*’ genotypes were growth-inhibited, but *StWRKY65* overexpression lines exhibited stronger adaptability: compared with NT controls, plant height increased by 26.52%–33.45%, plant fresh weight by 22.86%–27.55%, dry weight by 25.38%–30.41%, root fresh weight by 24.07%–32.33%, root dry weight by 26.29%, 36.85%, and tuber weight by 35.59%–47.64%. In contrast, RNAi lines showed decreases in plant height (26.29%–31.76%), plant fresh weight (29.62%–32.81%), dry weight (25.25%–30.45%), root fresh weight (23.9%–30.25%), root dry weight (26.18%–31.53%), and tuber weight (35.33%–49.89%) compared with NT controls (**Figures 4A–F**). Under severe 35°C heat stress, the thermoprotective effect of *StWRKY65* in ‘*Atlantic*’ became more pronounced: OE lines displayed remarkable stress resistance, with plant height increased by 51.10%–64.90%, plant fresh and dry weights by 49.38%–57.67% and 36.11%–36.93%, respectively, root fresh and dry weights by 46.42%–63.97% and 43.15%–57.38%, respectively, and tuber weight by 77.56%–97.53% relative to NT controls. Conversely, RNAi lines were highly sensitive to heat stress, showing drastic reductions in plant height (51.38%–61.70%), plant fresh and dry weights (57.77%–65.16% and 50.61%–59.24%), root fresh and dry weights (46.53%–56.27% and 43.28%–57.82%), and tuber weight (92.32%–94.91%) (**Figures 4A–F**), compared to NT lines. Similar trends were observed in ‘*Desiree*’ plants under heat stress: at 30°C, *StWRKY65*-overexpression lines showed increases in plant height (26.70%–32.47%), plant fresh weight (26.35%–33.14%), plant dry weight (24.61%–30.12%), root fresh weight (25.58%–35.22%), root dry weight (29.50%–37.48%), and tuber weight (45.49%–56.31%) compared with NT controls, while RNAi lines exhibited decreases in these parameters (26.32%–32.03% for plant height, 27.01%–34.85% for plant fresh weight, 25.11%–32.74% for plant dry weight, 25.54%–32.52% for root fresh weight, 29.77%–36.34% for root dry weight, and 46.58%–57.86% for tuber weight) (**Figures 5A–F**). At 35°C, OE lines further demonstrated enhanced heat tolerance (plant height +39.12%–49.66%, plant fresh/dry weights +40.37%–48.89%/+38.06%–53.09%, root fresh/dry weights +40.32%–50.97%/+48.52%–65.38%, tuber weight +79.54%–98.74%), whereas RNAi lines suffered severe growth inhibition (plant height −38.69%–46.15%, plant fresh/dry weights −38.86%–47.60%/−39.55%–41.60%, root fresh/dry weights −41.29%–50.94%/−49.17%–61.71%, tuber weight −78.90%–95.60%), compared to NT potato lines (**Figures 5A–F**). Collectively, these results indicate that *StWRKY65* enhances potato adaptability and yield stability under heat stress by regulating relevant growth indices, with OE lines consistently outperforming NT and RNAi lines across different temperatures (30°C and 35°C) and varieties.

### 3.6 *StWRKY65* enhances thermotolerance in potato by regulating antioxidant defense, osmoprotectant accumulation, and chlorophyll content under heat stress

**Figure 6:**
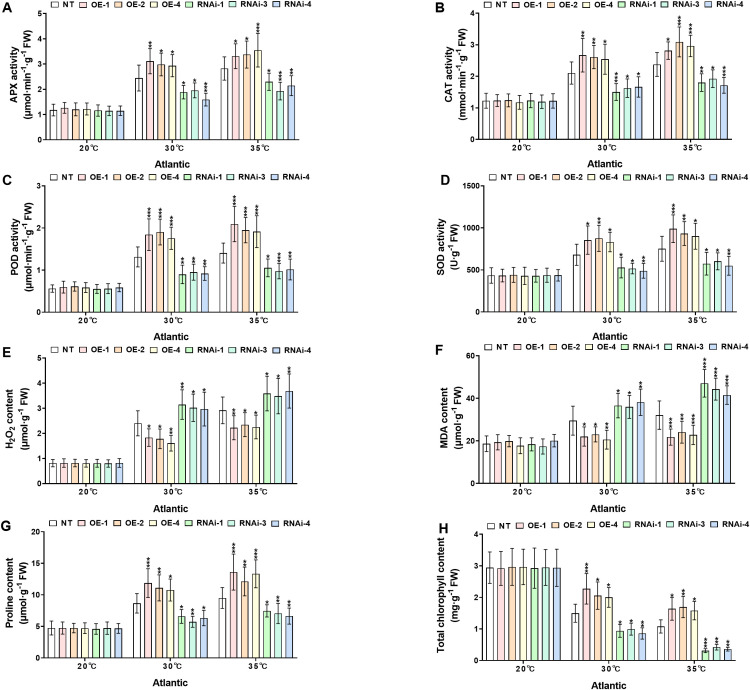
*StWRKY65* modulates potato physiological indices of cultivar ‘*Atlantic*’; **(A)** APX activity, **(B)** CAT activity, **(C)** POD activity, **(D)** SOD activity, **(E)** H_2_O_2_ content, **(F)** MDA content, **(G)** Proline content, and **(H)** Total chlorophyll content, after exposure to 20°C, 30°C, and 35°C of heat stress treatments. NT, non-transgenic plants; OE, pBI121-EGFP-StWRKY65-transgenic plants (OE-1, OE-2, and OE-4); RNAi, pART-StWRKY65-RNAi-transgenic plants (RNAi-1, RNAi-3 and RNAi-4). The data are presented as mean ± standard deviation. P-values (*P < 0.05, **P < 0.01, ***P < 0.001) were calculated through ordinary two-way ANOVA followed by Tukey’s multiple comparisons test with a sample size of n = 9.

**Figure 7:**
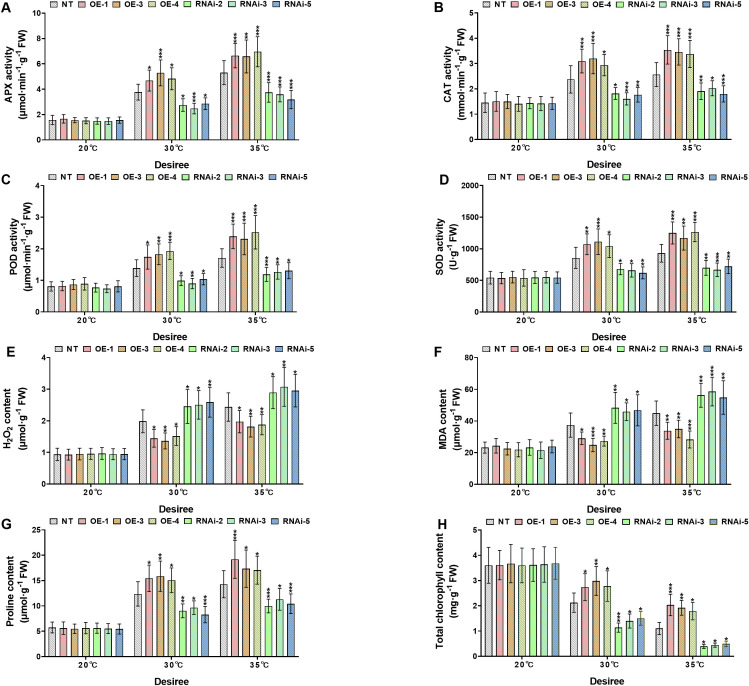
*StWRKY65* modulates potato physiological indices of cultivar ‘*Desiree*’; **(A)** APX activity, **(B)** CAT activity, **(C)** POD activity, **(D)** SOD activity, **(E)** H_2_O_2_ content, **(F)** MDA content, **(G)** Proline content, and **(H)** Total chlorophyll content, after exposure to 20°C, 30°C, and 35°C of heat stress treatments. NT, non-transgenic plants; OE, pBI121-EGFP-StWRKY65-transgenic plants (OE-1, OE-3, and OE-4); RNAi, pART-StWRKY65-RNAi-transgenic plants (RNAi-2, RNAi-3 and RNAi-5). The data are presented as mean ± standard deviation. P-values (*P < 0.05, **P < 0.01, ***P < 0.001) were calculated through ordinary two-way ANOVA followed by Tukey’s multiple comparisons test with a sample size of n = 9.

To investigate the physiological responses of transgenic and NT potato lines of the cultivars ‘*Atlantic*’ and ‘*Desiree*’ under different temperature stress conditions (20°C, 30°C, and 35°C), the key antioxidant enzymes (APX, CAT, POD, SOD), oxidative stress marker (H_2_O_2_ and MDA), osmoprotectant proline, and total chlorophyll content were compared between NT and transgenic lines (OE and RNAi), as shown in **Figures 6**, **7**. Under the control condition (20°C), no significant differences (*P* > 0.05) were observed in all the aforementioned physiological indicators between transgenic and NT plants. Under the conditions of 30°C and 35°C, the activities of APX (**Figures 6**, **7A**), CAT (**Figures 6**, **7B**), POD (**Figures 6**, **7C**), and SOD (**Figures 6**, **7D**) in the OE lines were significantly higher than those in the NT and RNAi lines (*P < 0.05, **P < 0.01, ***P < 0.001), indicating that the overexpression of *StWRKY65* could enhance the antioxidant defense system and more efficiently scavenge reactive oxygen species (ROS) under heat stress. Meanwhile, the contents of oxidative stress markers H_2_O_2_ (**Figures 6**, **7E**) and MDA (**Figures 6**, **7F**) were also analyzed. Compared with the NT lines, the OE lines had lower contents while the RNAi lines had higher contents under high temperatures (30°C and 35°C) (*P < 0.05, **P < 0.01, ***P < 0.001), suggesting that the OE lines suffered less oxidative damage, and the RNAi lines were more vulnerable. In addition, the osmotic regulatory substance proline (**Figures 6**, **7G**) and total chlorophyll content (**Figures 6**, **7H**) were analyzed. The osmoprotectant proline was significantly accumulated in the OE lines under heat stress (30°C and 35°C), which was helpful for osmotic adjustment, while the accumulation of proline in the RNAi lines was relatively less (*P < 0.05, **P < 0.01, ***P < 0.001). The decrease in total chlorophyll content was smaller in the OE lines at 30°C and 35°C, indicating better maintenance of photosynthetic capacity, while the RNAi lines had more significant chlorophyll loss (*P < 0.05, **P < 0.01, ***P < 0.001). In conclusion, *StWRKY65* significantly improved the heat tolerance of potato by enhancing antioxidant defense, reducing oxidative damage, promoting the accumulation of osmotic regulatory substances, and decreasing the loss of total chlorophyll content to maintain photosynthetic integrity.

### 3.7 *StWRKY65* mitigates heat stress responses by modulating antioxidant-related gene networks in potato

**Figure 8:**
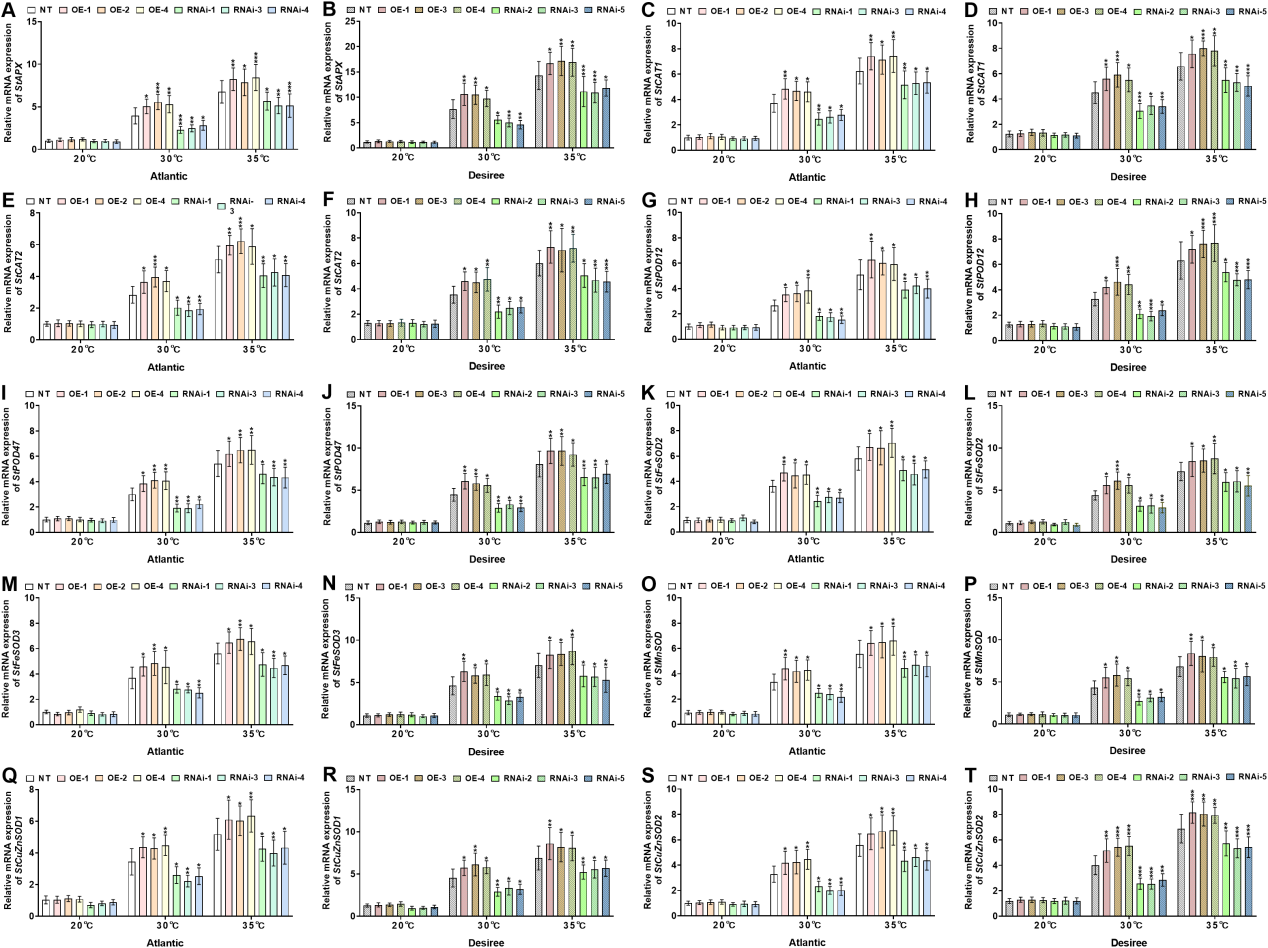
*StWRKY65* regulates the relative mRNA expression of potato plants of cultivar ‘*Atlantic*’ ‘*Desiree*’; **(A, B)** *StAPX*, **(C, D)** *StCAT1*, **(E, F)** *StCAT2*, **(G, H)** *StPOD12*, **(I, J)** *StPOD47*, **(K, L)** *StFeSOD2*, **(M, N)** *StFeSOD3*, **(O, P)** *StMnSOD*, **(Q, R)** *StCuZnSOD1*, and **(S, T)** *StCuZnSOD2*, respectively, after exposure to 20°C, 30°C, and 35°C of heat stress. In the ‘*Atlantic*’ cultivar: NT: non-transgenic plants; OE, pBI121-EGFP-StWRKY65-transgenic plants (OE-1, OE-2, and OE-4); RNAi, pART-StWRKY65-RNAi-transgenic plants (RNAi-1, RNAi-3, and RNAi-5). In the ‘*Desiree*’ cultivar: NT: non-transgenic plants; OE, pBI121-EGFP-StWRKY65-transgenic plants (OE-1, OE-3, and OE-4); RNAi, pART-StWRKY65-RNAi-transgenic plants (RNAi-2, RNAi-3, and RNAi-5). The data are presented as mean ± standard deviation. P-values (*P < 0.05, **P < 0.01) were calculated through ordinary two-way ANOVA followed by Tukey’s multiple comparisons test with a sample size of n = 9.

To further elucidate the role of *StWRKY65* in response to varying temperature stress conditions (20°C, 30°C, and 35°C), we analyzed the expression of stress-responsive genes in two potato cultivars (‘*Atlantic*’ and ‘*Desiree*’), including *StAPX*, *StCAT1*,​*StCAT2*, *tPOD12*, *StPOD47 StFeSOD2*, *StFeSOD3*,*​StMnSOD*, *StCuZnSOD1*, and *StCuZnSOD2*. As shown in **Figures 8A–T**, under 20°C, no significant differences in the relative mRNA expression levels of these antioxidant-related genes were observed between transgenic (OE and RNAi) and NT plants in either cultivar. Under 30°C heat stress, the *StWRKY65*-overexpressing lines (‘*Atlantic*’: OE-1/2/4; ‘*Desiree*’: OE-1/3/4) exhibited​significant upregulation of stress-responsive genes, including *StAPX* (**Figures 8A, B**), *StCAT1* (**Figures 8C, D**) *StCAT2* (**Figures 8E, F**), *StPOD12* (**Figures 8G, H**), *StPOD47* (**Figures 8I, J**), *StFeSOD2* (**Figures 8K, L**), *StFeSOD3* (**Figures 8M, N**), *StMnSOD* (**Figures 8O, P**), *StCuZnSOD1* (**Figures 8Q, R**), and *StCuZnSOD2* (**Figures 8S, T**), compared to NT controls. In contrast, the RNAi lines (‘*Atlantic*’: RNAi-1/3/4; ‘*Desiree*’: RNAi-2/3/5) showed significant downregulation of these genes under the same stress conditions. Similarly, under severe heat stress (35°C), the OE lines (‘*Atlantic*’: OE-1/2/4; ‘*Desiree*’: OE-1/3/4) displayed even more pronounced upregulation of the aforementioned stress-responsive genes (*StAPX*, *StCAT1*, *StCAT2*, *StPOD12*, *StPOD47*, *StFeSOD2*, *StFeSOD3*, *StMnSOD*, *StCuZnSOD1*, and *StCuZnSOD2*). Conversely, compared to NT controls, the RNAi lines (‘*Atlantic*’: RNAi-1/3/4; ‘*Desiree*’: RNAi-2/3/5) exhibited significant downregulation of these antioxidant-related genes. These findings demonstrate that *StWRKY65* plays a critical role in regulating multiple antioxidant enzyme genes under heat stress (30°C and 35°C), thereby mitigating oxidative stress responses induced by elevated temperatures.

### 3.8 *StWRKY65* modulates heat stress responses by regulating photosynthetic parameters in potatoes

**Figure 9:**
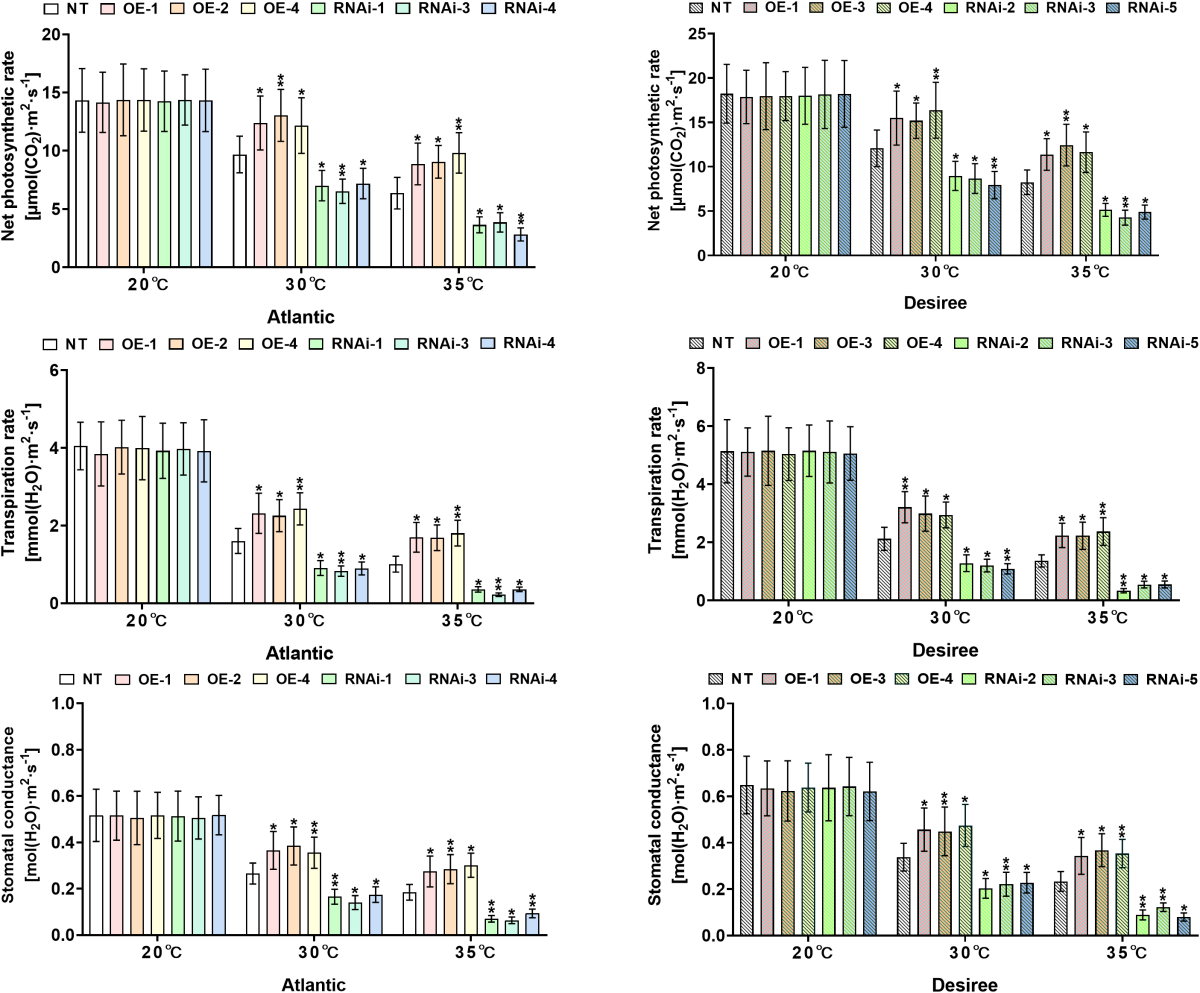
*StWRKY65* regulates the photosynthetic traits of potato plants of cultivars, ‘*Atlantic*’ and ‘*Desiree*’; **(A, B)** net photosynthetic rates, **(C, D)** transpiration rates, and **(E, F)** stomatal conductance, respectively, after exposure to 20°C, 30°C, and 35°C of heat stress treatments. In the ‘*Atlantic*’ cultivar: NT: non-transgenic plants; OE, pBI121-EGFP-StWRKY65-transgenic plants (OE-1, OE-2, and OE-4); RNAi, pART-StWRKY65-RNAi-transgenic plants (RNAi-1, RNAi-3, and RNAi-5). In the ‘*Desiree*’ cultivar: NT: non-transgenic plants; OE, pBI121-EGFP-StWRKY65-transgenic plants (OE-1, OE-3, and OE-4); RNAi, pART-StWRKY65-RNAi-transgenic plants (RNAi-2, RNAi-3, and RNAi-5). The data are presented as mean ± standard deviation. P-values (*P < 0.05, **P < 0.01) were calculated through ordinary two-way ANOVA followed by Tukey’s multiple comparisons test with a sample size of n = 9.

Analysis of gas exchange parameters revealed significant variations in both ‘*Atlantic*’ and ‘*Desiree*’ potato cultivars under different magnitudes of heat stress applications (20°C, 30°C, and 35°C). As shown in **Figures 9A–F**, under control conditions (20°C), there were no significant changes (*P*>0.05) in net photosynthetic rate, stomatal conductance, or transpiration rate between transgenic (OE lines and RNAi lines) and NT plants of both cultivars. Under 30°C heat stress, *StWRKY65*-overexpressing lines of both potato cultivars (‘*Atlantic*’: OE-1/2/4; ‘*Desiree*’: OE-1/3/4) exhibited significantly higher Pn (**Figures 9A, B**), Gs (**Figures 9C–E**), (**Figures 9E, F**) compared to NT plants, whereas RNAi lines (‘*Atlantic*’: RNAi-1/3/4; ‘*Desiree*’: RNAi-2/3/5) showed significant decreases in these gas exchange parameters. Similarly, under 35°C heat stress, all experimental plants of both cultivars experienced a reduction in photosynthetic efficiency, but *StWRKY65*-overexpressing lines (‘*Atlantic*’: OE-1/2/4; ‘*Desiree*’: OE-1/3/4) still maintained significantly higher Pn (**Figures 9A, B**), Gs (**Figures 9C–E**), (**Figures 9E, F**) than NT lines, while RNAi lines (‘*Atlantic*’: RNAi-1/3/4; ‘*Desiree*’: RNAi-2/3/5) showed further decreases in these photosynthetic parameters. The contrasting responses between overexpression and knockout genotypes strongly support the regulatory role of *StWRKY65* in maintaining photosynthetic efficiency under high-temperature stress (30°C and 35°C).

## 4 Discussion

WRKY TFs are widely distributed in plants, such as *Arabidopsis* (Li et al., 2010), tobacco (Zhang et al., 2024b), rice (Lee et al., 2018), tomato (Shang et al., 2024), wheat (He et al., 2016), and maize (Wang et al., 2018a), and play key regulatory roles in various life processes and stress responses as part of the plant stress signaling pathway (Jiang et al., 2017; Kang et al., 2021; Liu et al., 2024; Jiang et al., 2025). Despite the critical role of WRKY TFs in abiotic stress responses, their functional characterization in potato, the world’s fourth most important staple crop, remains poorly understood, particularly under heat stress conditions. Here, we investigated the function of StWRKY65 under different heat stresses through transgenic techniques to elucidate its regulatory role in thermotolerance. In this study, we initially investigated the sequence alignment analysis, which revealed that StWRKY65 contains a canonical WRKYGQK domain at its N-terminus, along with a C_2_H_2_-type zinc-finger motif (CX_5_-C-X_23_-H-X_1_-H), consistent with its classification as a Group II WRKY transcription factor (**Figure 1A**). Phylogenetic tree construction further supported this classification, grouping StWRKY65 with functionally related homologs from other plant species, suggesting evolutionary conservation of its regulatory role (**Figure 1B**). Our investigation is supported by a previous report, which explored that ZmWRKY65, a candidate WRKY TF identified from drought and SA-treated maize transcriptomic data, encodes a 516-amino acid protein featuring a conserved WRKYGQK domain, an N-terminal coiled-coil region (aa 102–142), and a C_2_H_2_ zinc-finger motif (CX_5_CX_23_ HX_1_H). Phylogenetic analysis (MEGA 5.1, neighbor-joining) of 15 homologs from diverse species and 14 maize WRKYs (Phytozome 12.1) revealed close evolutionary relationships between ZmWRKY65 and AtWRKY3 (*Arabidopsis*), OsWRKY96 (rice), SiWRKY3 (millet), and GmWRKY33 (soybean), suggesting functional conservation among them (Huo et al., 2021).

WRKY TFs typically exhibit tissue-specific expression patterns and are influenced by environmental stresses, enabling them to regulate diverse biological processes in plants. For example, in pepper (*Capsicum annuum*), *CaWRKY40* is upregulated under heat stress and acts as a positive regulator of thermotolerance (Liu et al., 2021b). To further elucidate the role of *StWRKY65* in thermotolerance, we analyzed its expression profile in the leaf tissues of two potato cultivars, *Atlantic* and *Desiree* (**Figures 2A–L**). qRT-PCR-based expression profiling revealed cultivar-specific differential regulation of *StWRKY65* under heat stress (30°C and 35°C) at multiple time points. Notably, both cultivars exhibited peak transcript accumulation at 48 h post-induction, suggesting a time-dependent regulatory response to elevated temperatures. These findings establish *StWRKY65* as a potential mediator of heat stress adaptation in potato. Our study showed a similar research trend to previous research on *Pinellia ternata* that revealed tissue-specific expression of five *PtWRKY* genes, with *PtWRKY1/3/4/5* exhibiting the highest expression in roots and *PtWRKY2* in leaves. Under heat stress, only *PtWRKY2* was strongly induced (200-fold at 12 h), while others were suppressed, suggesting its key role in thermotolerance (Liu et al., 2024). As TFs, WRKY proteins are predominantly localized in the nucleus and exhibit transcriptional regulatory activity (Gong et al., 2015; Jiang et al., 2017). Subcellular localization analysis demonstrated nuclear localization of the StWRKY65 protein, as evidenced by distinct GFP fluorescence signals exclusively detected in the nucleus (**Figure 3**). In contrast, control vectors expressing free GFP exhibited diffuse fluorescence patterns distributed across the plasma membrane, cytoplasm, and nuclear compartments. This observation confirms the nuclear targeting capability of *StWRKY65*, consistent with its predicted function as a TF. The BrWRKY65-GFP fusion protein was transiently expressed in *Nicotiana benthamiana* epidermal cells. Confocal microscopy showed nuclear-specific GFP fluorescence, co-localizing with DAPI-stained nuclei, while free GFP displayed diffuse cytoplasmic and nuclear distribution. These results confirm *BrWRKY65*’s nuclear localization, consistent with our investigation (Fan et al., 2017).

Abiotic stress, such as extreme temperatures, disrupts plants’ key physiological and biochemical processes, ultimately hindering growth and biomass production. To counteract these challenges, plants activate sophisticated defense mechanisms or key genes, which modulate growth patterns and restore metabolic pathways to maintain cellular homeostasis and ensure survival (Zhang et al., 2020). Our study investigated the role of the *StWRKY65* gene in enhancing thermotolerance in potato plants under heat stress (30°C and 35°C). Remarkably, *StWRKY65-*overexpressing plants exhibited a significant increase in plant height, tuber yield, total fresh and dry biomass, and root systems compared to NT controls, as recorded in **Figures 4**, **5A–F**. Conversely, RNAi-silenced plants showed a decline in these growth parameters, confirming the gene’s critical role in heat stress resilience. Notably, under optimal temperatures, all experimental plants performed equally, supporting that *StWRKY65* specifically functions in thermotolerance rather than baseline growth. In a previous report, after mannitol (200–300 mM) stress application, lateral root number remained unchanged, but root length decreased in WT and OE lines. However, *MfWRKY40*-OE lines A and C maintained longer roots (1.19–1.75-fold of WT). Similarly, under NaCl (50–100 mM) stress application, *MfWRKY40*-OE lines A and C showed significantly longer primary roots (1.21–1.50-fold of WT) (Huang et al., 2022). Similarly, another study has investigated the heterologous overexpression of *IlWRKY70* in *Nicotiana tabacum*, which confers enhanced tolerance to NaCl-induced salinity and drought stress, as demonstrated by significantly improved germination rates, increased root elongation, and biomass accumulation (fresh weight) relative to the WT controls (Shi et al., 2023). Importantly, the increase in fresh weight aligns with our findings.

Under heat stress, antioxidant enzymes and oxidative markers are critical for maintaining cellular homeostasis. Enzymes such as SOD, CAT, POD, and glutathione peroxidase (GPX) are upregulated to neutralize ROS and mitigate oxidative damage. Meanwhile, oxidative markers like MDA, H_2_O_2_, and reactive nitrogen species (RNS) serve as indicators of oxidative stress levels. A precise equilibrium between ROS generation and antioxidant activity is vital; however, heat stress can disrupt this balance, leading to excessive ROS accumulation and subsequent oxidative damage (Habashy et al., 2019). Furthermore, Osmolytes, such as proline, serve as key signaling molecules in enhancing plant heat stress tolerance (Iqbal et al., 2019). Our study revealed that under heat stress (30°C and 35°C), *StWRKY65*-overexpressing potato plants (cultivars *Atlantic* and *Desiree*) exhibited increased antioxidant enzyme activity (APX, POD, CAT, SOD), higher proline and chlorophyll levels, and reduced oxidative damage (lower MDA and H_2_O_2_) compared to NT plants (**Figures 6**, **7A–F**). In contrast, *StWRKY65* RNAi lines showed the opposite trend. No differences were observed between transgenic and NT lines under control conditions (20°C). Our findings under heat stress showed similar research trend to a previous research under cold and drought stress, which described that the overexpression of the *Malus baccata* WRKY TF, *MbWRKY65* increased drought and freezing tolerance in transgenic tomatoes by reducing oxidative stress markers (MDA, H_2_O_2_, O_2_^-^) and enhancing proline, chlorophyll, and antioxidant enzyme activity (Yu et al., 2024). Additionally, *StWRKY65*-overexpressing plants exhibited significant upregulation of stress-responsive genes (*StAPX, StCAT1, StCAT2, StPOD12, StPOD47, StFeSOD2, StFeSOD3, StMnSOD, StCuZnSOD1*, and *StCuZnSOD2*) under heat stress conditions (30°C and 35°C). Conversely, RNAi-mediated suppression of *StWRKY65* resulted in downregulation of these genes compared to NT controls (**Figures 8A–T**). These findings suggest that *StWRKY65* plays a pivotal role in modulating antioxidant-related gene expression during heat stress. A prior study exhibited the functional analysis of *TaWRKY31*, which revealed contrasting expression patterns of antioxidant genes under drought stress conditions. Virus-induced gene silencing (VIGS) of *TaWRKY31* in wheat (BSMV: WRKY31-1as and BSMV: WRKY31-2as lines) significantly suppressed the expression of ROS scavenging genes, including *TaSOD* (Fe), *TaPOD*, and *TaCAT*. Conversely, heterologous overexpression of *TaWRKY31* in *Arabidopsis thaliana* enhanced transcript levels of orthologous antioxidant genes (*AtSOD* (Cu/Zn), *AtPOD*, and *AtCAT*) (Ge et al., 2024), which are consistent with our heat stress findings; these results suggest a universal stress adaptation role. Moreover, in Chinese cabbage, *BaWRKY22* activated and enhanced the *BaCAT2* transcript level and reduced H_2_O_2_ contents under heat stress (Wang et al., 2023), and overexpression of the *PtWRKY2* gene up-regulated the *POD34, CAT1*, and *SOD1* genes in transgenic *Arabidopsis* under heat stress (Liu et al., 2024). As a transcription factor, StWRKY65 may directly or indirectly regulate these antioxidant genes or other regulatory factors, thereby increasing the expression of corresponding proteins and enzyme activity. This enhances the efficient scavenging of ROS (evidenced by reduced H_2_O_2_ and MDA levels), protects cellular structures (maintaining membrane stability and photosynthetic pigments), and ultimately promotes growth and yield formation under heat stress.

Extreme heat stress disrupts the photosynthetic apparatus in plant cells, leading to a significant decline in photosynthetic efficiency (Mathur et al., 2014). Our findings reveal that sustained exposure to elevated temperatures (30–35°C) induced significant photosynthetic impairment in both RNAi and NT potato plants. This thermal stress disrupted photosynthetic performance through a cascade of interconnected physiological distresses, including a marked decline in photosynthetic efficiency, suppression of transpiration rates, and pronounced stomatal dysfunction, as evidenced by aberrant stomatal conductance and aperture regulation. On the other hand, *StWRKY65*-overexpressing potato plants in both cultivars (‘*Atlantic’* and ‘*Desiree’*) under varying heat stress conditions (30°C and 35°C) exhibited significantly higher photosynthetic rate, transpiration rate, and stomatal conductance (**Figures 9A–F**), which maintained stomatal aperture, preserve chloroplast functions, improve water moment, and preserve ROS damage. Under optimal growth conditions (20°C), no significant differences were observed between transgenic (OE and RNAi) and NT plants, confirming that *StWRKY65*-mediated thermotolerance is stress-inducible. A previous report unveiled that the high-temperature stress primarily targets Photosystem II (PSII) and Rubisco, with additional effects on Cytochrome b559 (Cytb559) and plastoquinone (PQ). Key responses include ROS accumulation, heat shock protein production, and altered secondary metabolite synthesis (Mathur et al., 2014). Additionally, high temperatures damage the chloroplast Structure, such as thylakoid membrane destabilization, which leads to unstacking of grana and reduced light-harvesting efficiency. Furthermore, stroma lamellae dilation disrupts electron transport chain organization, and lipid peroxidation degrades membrane integrity due to ROS accumulation (Dutta et al., 2009). These findings align with previous research demonstrating that *AtWRKY30* overexpression in wheat enhances gas exchange parameters (Pn, Gs, E) and relative water content (RWC) under combined heat and drought stress. In that study, transgenic wheat lines maintained superior photosynthetic efficiency and water retention compared to WT plants under stress, while no genotypic differences were detected under control conditions (El-Esawi et al., 2019). It can be further elaborated that maintaining better photosynthetic capacity (by protecting photosynthetic machinery and stomatal function) not only provides energy and carbon skeletons but may also reduce ROS production from photo-inhibition and photorespiration, forming a synergistic effect with antioxidant defense. Conversely, an enhanced antioxidant system can also protect photosynthetic structures from oxidative damage. Collectively, these results suggest that WRKY TFs (*StWRKY65* and *AtWRKY30*) play a conserved role in mitigating heat-induced photosynthetic and antioxidant defense inhibition by regulating stomatal behavior, cellular water status, and biochemical processes.

This study provides valuable insights into the role of *StWRKY65* in potato thermotolerance, yet several noteworthy limitations warrant attention. First, while the research demonstrates *StWRKY65*’s regulatory effects on antioxidant gene expression and physiological responses under heat stress, the specific molecular mechanisms underlying its interaction with downstream target gene promoters, including the identification of precise cis-acting elements and coregulatory proteins, remain unclear. Further investigations, such as chromatin immunoprecipitation (ChIP)-seq or yeast one-hybrid assays, are needed to validate direct binding events and regulatory networks. Second, the current analysis focused solely on seedlings under controlled growth chamber conditions. The translational relevance of *StWRKY65* overexpression in field-scale heat stress scenarios, incorporating temperature fluctuations and combined environmental stresses like drought and high light, has not been explored. Multi-season and multi-location field trials are essential to evaluate the stability and practical applicability of the observed thermotolerant phenotypes. Additionally, although the study assessed growth parameters and oxidative stress markers in two potato cultivars, the generalizability of *StWRKY65*-mediated thermotolerance across diverse genetic backgrounds (e.g., wild potato species or other crops) requires validation. Expanding the study to include more genotypes would enhance the ecological and agronomic significance of the findings. Finally, while the study highlights *StWRKY65*’s role in ROS scavenging, its potential crosstalk with other stress-signaling pathways (e.g., abscisic acid, calcium signaling) or metabolic networks (e.g., carbohydrate allocation) remains underexplored. Integrated omics approaches (transcriptomics, proteomics, metabolomics) could unveil broader regulatory landscapes and functional synergies, offering a more comprehensive understanding of heat stress adaptation mechanisms in potato. These limitations underscore the need for follow-up studies to elucidate molecular mechanisms, validate field performance, expand genetic diversity, and explore multi-pathway interactions, thereby enhancing the translational application value of *StWRKY65* in crop improvement.

## 5 Conclusion

This research highlights the essential function of *StWRKY65*, a Group II WRKY transcription factor localized in the nucleus, in improving thermotolerance in potato. Overexpression of *StWRKY65* markedly improves thermotolerance by enhancing the activities of key antioxidant enzymes (APX, CAT, POD, SOD), increasing proline levels, maintain chlorophyll content, optimizing photosynthetic parameters (including photosynthesis, transpiration, and stomatal conductance), and decreasing H_2_O_2_ and MDA accumulation. Additionally, the overexpression of *StWRKY65* was shown to transcriptionally activate important genes associated with antioxidant enzymes, including *StAPX, StCAT1, StCAT2, StPOD12, StPOD47, StFeSOD2, StFeSOD3, StMnSOD, StCuZnSOD1*, and *StCuZnSOD2*. In contrast, plants with *StWRKY65* knockdown show reduced thermotolerance and adverse physiological responses. These results position *StWRKY65* as a promising target for molecular breeding aimed at enhancing heat resilience in potato and related species, thereby providing a sustainable approach to address the challenges posed by climate change on agricultural productivity.

## Data Availability

The datasets presented in this study can be found in online repositories. The names of the repository/repositories and accession number(s) can be found in the article/**Supplementary Material**.
