# Supplementary material for: StWRKY65 stimulates thermotolerance in potato (Solanum tuberosum L.) through antioxidant and photosynthetic modulation
Source: Front Plant Sci. 2025 Jul 21;16:1634338. doi: 10.3389/fpls.2025.1634338 (PMC12319013; doi:10.3389/fpls.2025.1634338)
Supplement: Supplementary file 1 [file Table1.docx]

**Title: *StWRKY65* Enhances Thermotolerance in Potato (*Solanum tuberosum* L.) by Orchestrating Antioxidant Defense, Photosynthetic Efficiency, and Growth Regulation under Heat Stress**

**Supplementary Materials**

1. **Supplementary Figures**


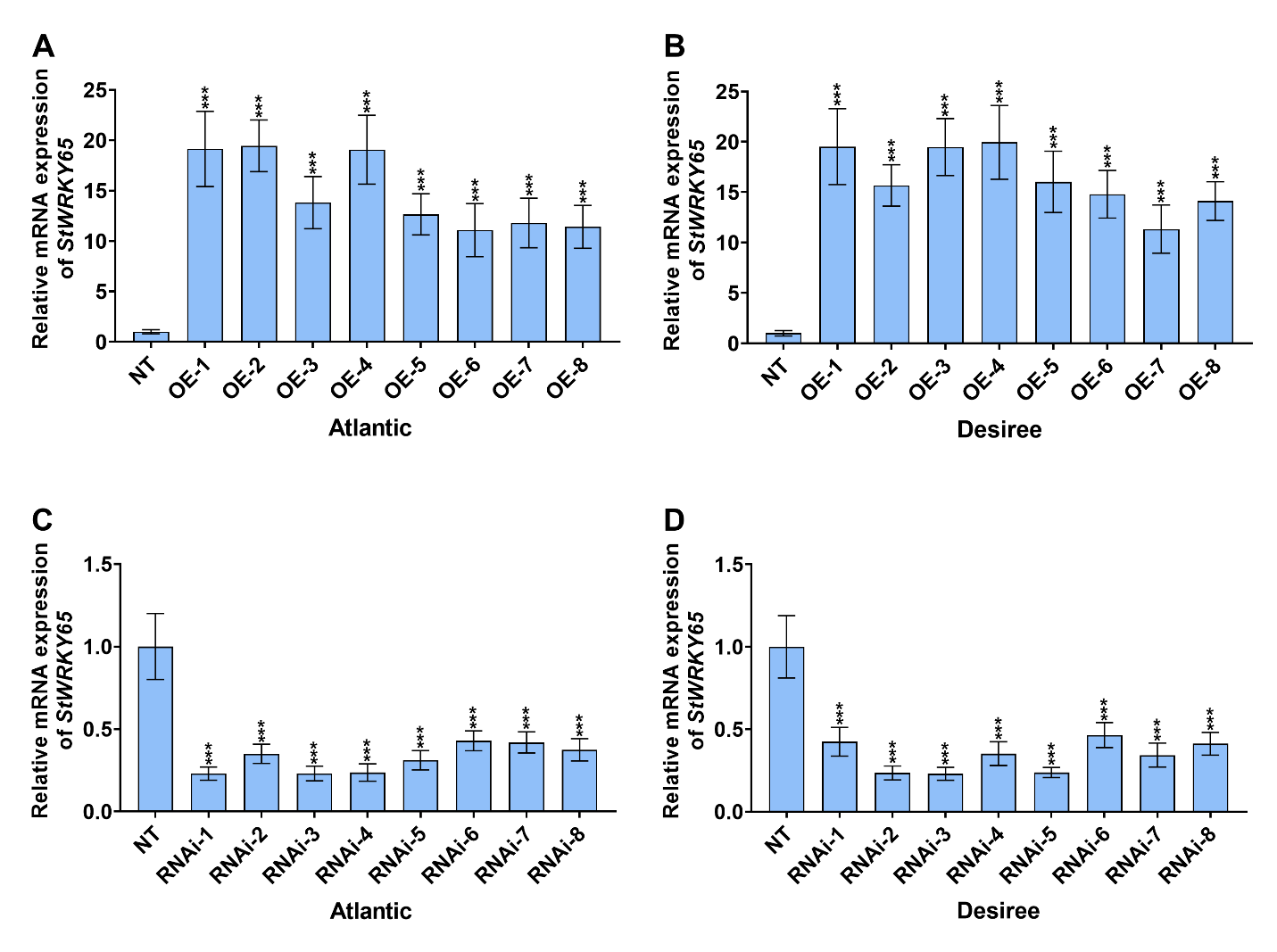


**Figure S1**. The relative mRNA expression levels of *StWRKY65* in two potato cultivars: (A and B) *StWRKY65* overexpression lines; (C and D) RNAi knockdown lines. OE, pBI121-EGFP-StWRKY65-transgenic plants (OE-1, OE-2, OE-3, OE-4, OE-5, OE-6, OE-7, and OE-8); RNAi, pART-StWRKY65-RNAi-transgenic plants (RNAi-1, RNAi-2, RNAi-3, RNAi-4, RNAi-5, RNAi-6, RNAi-7 and RNAi-8). In the ​​‘*Desiree*’​​ cultivar: NT​​: non-transgenic plants; OE, pBI121-EGFP-StWRKY65-transgenic plants (OE-1, OE-2, OE-3, OE-4, OE-5, OE-6, OE-7, and OE-8); RNAi, pART-StWRKY65-RNAi-transgenic plants (RNAi-1, RNAi-2, RNAi-3, RNAi-4, RNAi-5, RNAi-6, RNAi-7, and RNAi-8). The data are presented as mean ± standard deviation. P-values (****P < 0.001*) were calculated through ordinary two-way ANOVA followed by Tukey’s multiple comparisons test with a sample size of n = 9.

1. **Supplementary Tables**

**Table S1.** Protein IDs of the *WRKY65* transcription factor in different plant species.

| **Plant species** | **Protein** | **Protein ID** |
| --- | --- | --- |
| *Arabidopsis thaliana* | AtWRKY65 | NP_174222.2 |
| *Solanum tuberosum* | StWRKY65 | XP_006348892.1 |
| *Solanum lycopersicum* | SlWRKY65 | XP_010323672.1 |
| *Capsicum annuum* | CaWRKY65 | XP_016580885.2 |
| *Lycium ferocissimum* | LfWRKY65 | XP_059283269.1 |
| *Solanum dulcamara* | SdWRKY65 | XP_055824776.1 |
| *Nicotiana tabacum* | NtWRKY65 | XP_016462409.1 |
| *Lycium barbarum* | LbWRKY65 | XP_060189713.1 |
| *Ipomoea batatas* | IbWRKY65 | GMD91537.1 |

The above-mentioned proteins can be directly accessed through the National Center for Biotechnology Information (NCBI database) (<https://www.ncbi.nlm.nih.gov/guide/>).

**Table S2.** Sequences of primers used in the present study.

| **Gene ID** | **Gene** | **Forward (5’-3’)** | **Reverse (5’-3’)** |
| --- | --- | --- | --- |
| **qRT-PCR:** | | | |
| XM_006347752.2 | *StEf1α* | GGTTGTATCTCTTCCGATAAAGGC | GGTTGTATCTCTTCCGATAAAGGC |
| AB041343 | *StAPX* | CTCCTCTGTGATCCTGCTTTC | GAGAGTGTCAAGTGAGCCTTAG |
| AY442179 | *StCAT1* | GCACAGGGATGAGGAGATCG | CTTCTCACGTTTGCCACTGC |
| XM_006340770.2 | *StCAT2* | GCACAGGGATGAGGAGATCG | CTTCTCACGTTTGCCACTGC |
| XM_006358985.2 | *StPOD12* | CGGCCTTCTTCGTCTTCACT | AAACGACTCTACCGCAGTCC |
| XM_006350750.2 | *StPOD47* | AGTCTGAGCAGGCCTTTGAC | GCCCATTTTACGCATGGCTT |
| XM_006358116.2 | *StFeSOD2* | GCAGCCAAATTCAGCACACT | GGACCAGCTTTCCTCGCTAA |
| XM_006350307.2 | *StFeSOD3* | TGCTGCCCAGGTATGGAATC | CCTCTCTGCTCAAGACGAGC |
| XM_006358693.2 | *StMnSOD* | TAGACGTTTGGGAACACGCA | CTCTTCAGGGGCACTCGTTT |
| XM_049521383.1 | *StCuZnSOD1* | CCTCCAACAGGTCACTGCTC | TCAGGTCACCCTTGAATGGC |
| AF354748 | *StCuZnSOD2* | TGTGGCACCATCCTCTTCAC | TCCTGTTGACATGCAGCCAT |
| XM_006348830.2 | *StWRKY65* | GGAGACAACAATGCAACGGC | ACGTGGATCCCACCATTGAC |

The above-mentioned genes can be directly accessed through the National Center for Biotechnology Information (NCBI database) ([https://www.ncbi.nlm.nih.gov/guide/](https://www.ncbi.nlm.nih.gov/guide/)).

1. **Supplementary data 1**
   1. **Assessment of physiological indicators**

The following physiological indicators were measured as listed below.

- - 1. **Antioxidant enzyme activity determination**

For enzyme extraction, 200 mg of frozen leaf tissue was mechanically homogenized in 4 mL of chilled 50 mM potassium phosphate buffer (pH 7.0). The extraction buffer contained 2 mM sodium EDTA and 1% (w/v) polyvinylpyrrolidone (PVPP) to enhance protein stability and remove phenolic compounds. Following homogenization, the samples were centrifuged at 10,000 × g for 10 minutes at 4°C to pellet cellular debris. The clarified supernatants were carefully transferred to fresh micro-centrifuge tubes and immediately stored at -80°C to preserve enzyme activity until analysis. To ensure experimental reproducibility, all enzymatic assays were performed using independently prepared extracts from three separate biological replicates. Enzyme activity measurements were conducted within two weeks of sample preparation to minimize degradation effects.

- - 1. **Ascorbate peroxidase (APX) activity**

The enzymatic activity of APX was evaluated by monitoring the oxidation rate of ascorbic acid at 290 nm wavelength, following the methodology established by Nakano and Asada (1981). The assay system (1 mL total volume) contained the following components, including 50 mM potassium phosphate buffer (pH 7.0), 1 mM EDTA-Na₂ (as a stabilizing agent), 0.5 mM freshly prepared ascorbic acid (substrate), 0.1 mM hydrogen peroxide (H₂O₂, reaction initiator), and 25 µL of enzyme extract. The reaction was started by adding H₂O₂, and the decrease in absorbance was recorded for 2 minutes at 25°C. APX activity was calculated using the molar extinction coefficient of 2.8 mM⁻¹ cm⁻¹ for oxidized ascorbate. One unit of APX activity was defined as the amount of enzyme required to oxidize 1 μmol of ascorbate per minute under the specified assay conditions.

- - 1. **Peroxidase (POD) activity**

POD activity was determined spectrophotometrically by measuring the oxidation of guaiacol at 470 nm, following the established protocol (Maehly and Chance, 1954). The 3 mL reaction system contained 2.84 mL of 10 mM potassium phosphate buffer (pH 7.0), 50 µL of 20 mM guaiacol solution, and 90 µL of enzyme extract. The enzymatic reaction was initiated by adding 20 µL of 40 mM H₂O₂. The increase in absorbance was monitored for 2 minutes at 25°C. POD activity was measured using the guaiacol extinction coefficient of 26.6 mM⁻¹ cm⁻¹, with one enzyme unit defined as the amount required to produce 1 μmol of tetra-guaiacol per minute under the specified conditions.

- - 1. **Superoxide dismutase (SOD) activity**

SOD activity was determined by measuring the inhibition of nitroblue tetrazolium (NBT) reduction in a photochemical reaction, adapted from Giannopolitis and Ries (1977). The 3 mL reaction mixture contained 50 mM potassium phosphate buffer (pH 7.8), 0.1 mM EDTA (pH 8.0), 14.9 mM methionine, 63 µM NBT, 90 µL of enzyme extract, and 8 µM riboflavin. The reaction was initiated by exposing the mixture to 4,000 lux light for 5 min, followed by immediate transfer to darkness to stop the reaction. Two control reactions were included: one kept in complete darkness and another exposed to light. The absorbance was recorded at 560 nm using a spectrophotometer. One unit (U) of SOD activity was defined as the enzyme quantity causing 50% inhibition of NBT reduction under the assay conditions.

- - 1. **Catalase (CAT) activity**

CAT enzyme activity was measured spectrophotometrically by monitoring the decrease in absorbance at 240 nm resulting from H₂O₂ decomposition, according to the method described by Aebi (1984). The assay mixture contained 2.67 mL of 50 mM phosphate buffer (pH 7.0), to which 30 µL of enzyme extract and 300 µL of 100 mM H₂O₂ were added to initiate the reaction. The change in absorbance was recorded for 2 minutes at 25°C. CAT activity was determined using the molar extinction coefficient of 39.4 mM⁻¹ cm⁻¹ for H₂O₂, with one unit of enzyme activity defined as the amount required to decompose 1 μmol of H₂O₂ per minute under the assay conditions.

- - 1. **Proline content determination**

The proline concentration in leaf tissues was measured using an optimized protocol based on the method by Bates et al. (1973). Leaf samples were collected 48 hours following stress treatment for analysis. The extraction procedure involved sample preparation (Fresh leaf tissue (200-300 mg) was homogenized in 2 mL of 3% (w/v) sulfosalicylic acid and the homogenate was centrifuged at 10,000 × g for 20 minutes at 4°C to remove cellular debris), colorimetric reaction (1 mL of supernatant was mixed with 1 mL of acid-ninhydrin reagent, containing 1.25 g ninhydrin dissolved in 30 mL glacial acetic acid and 20 mL 6 M phosphoric acid, and the mixture was heated at 100°C for 60 minutes in a water bath), proline extraction (The reaction was terminated by rapid cooling on ice, proline was extracted into 2 mL toluene by vigorous vortexing for 15 seconds, and the organic phase was separated after 60 minutes of dark incubation at 25°C), and spectrophotometric analysis (Absorbance of the toluene phase was measured at 520 nm using toluene as blank and proline concentration was determined from a standard curve [0-20 µg/Ml]). Results were expressed as micrograms per gram fresh weight (µg·g⁻¹ FW) with standard error (n=3).

- - 1. **Malondialdehyde (MDA) content determination**

The extent of lipid peroxidation in leaf tissues was determined through MDA measurement, following an adapted thiobarbituric acid (TBA) assay protocol (Heath and Packer, 1968). Samples were collected after 48 hours of stress treatment from the upper fully expanded leaves, with three biological replicates analyzed (two leaves per plant averaged for each replicate). The experimental procedure involved the sample preparation (Fresh leaf tissue [200-300 mg] was homogenized in 2 mL of 0.1% (w/v) TCA and the homogenate was centrifuged at 10,000 × g for 20 minutes at 4°C), TBA reaction (1.8 mL of supernatant was combined with 2 mL of TBA reagent [0.5% TBA in 20% TCA], the mixture was heated at 100°C for 30 minutes in a water bath, and Samples were immediately cooled on ice for 10 minutes to stop the reaction), spectrophotometric analysis (After centrifugation at 10,000 × g for 5 minutes, the supernatant was analyzed, absorbance readings were taken at 532 nm [primary peak] and 600 nm [background correction], and a reagent blank [0.5% TBA in 20% TCA] was used for baseline correction), and calculation (MDA concentration was determined using the following equation:

MDA (μmol·g⁻¹ FW) = [(A₅₃₂ - A₆₀₀)/155,000] × V × (1/W)
where: 155,000 = molar extinction coefficient (M⁻¹cm⁻¹)
V = total volume (mL)
W = fresh tissue weight (g).

- - 1. **H_2_O_2_ content**

H_2_O_2_ was examined according to a previous method reported by Bouaziz et al. (2015) with modifications. Briefly, the leaves (1 g) were digested in 2 mL of 0.1% trichloroacetic acid, followed by centrifugation (12,000 rpm, 15 min). Then, 0.5 mL of supernatant was incubated with 0.5 mL of potassium phosphate (10 mM, pH 7.0) and 1 mL of potassium iodide (1 mol/L). The absorbance was examined at 390 nm.

- - 1. **Total chlorophyll content**

Total chlorophyll content was measured using a previously described method by Zhu et al. (2023). Total chlorophyll content in potato leaves was examined using the commercial chlorophyll assay 165 kit according to the manufacturer’s instructions (Item No Cat#BC0990; Solarbio, Beijing, China). Fresh potato leaves were collected and washed in distilled water. After draining the surface of the leaves, the midrib of the leaves was removed and cut into pieces. Approximately 0.1 g of leaves were weighed and ground thoroughly in 1 mL of water in the dark, which was then entirely transferred into a 10-mL volumetric flask, diluted with water to volume, and mixed. The volumetric flask was maintained in the dark for 3 h. The absorbance of the supernatant was measured at a wavelength of 663 nm and 645 nm using a spectrophotometer model (Perkin Elmer, Shelton, CT, USA).
